# A bacterial toxin-antitoxin module is the origin of inter-bacterial and inter-kingdom effectors of *Bartonella*

**DOI:** 10.1371/journal.pgen.1007077

**Published:** 2017-10-26

**Authors:** Alexander Harms, Marius Liesch, Jonas Körner, Maxime Québatte, Philipp Engel, Christoph Dehio

**Affiliations:** 1 Focal Area Infection Biology, Biozentrum, University of Basel, Basel, Switzerland; 2 Department of Fundamental Microbiology, University of Lausanne, Lausanne, Switzerland; University of Geneva Medical School, SWITZERLAND

## Abstract

Host-targeting type IV secretion systems (T4SS) evolved from conjugative T4SS machineries that mediate interbacterial plasmid transfer. However, the origins of effectors secreted by these virulence devices have remained largely elusive. Previous work showed that some effectors exhibit homology to toxins of bacterial toxin-antitoxin modules, but the evolutionary trajectories underlying these ties had not been resolved. We previously reported that FicT toxins of FicTA toxin-antitoxin modules disrupt cellular DNA topology via their enzymatic FIC (filamentation induced by cAMP) domain. Intriguingly, the FIC domain of the FicT toxin VbhT of *Bartonella schoenbuchensis* is fused to a type IV secretion signal–the BID (Bep intracellular delivery) domain—similar to the *Bartonella* effector proteins (Beps) that are secreted into eukaryotic host cells via the host-targeting VirB T4SS. In this study, we show that the VbhT toxin is an interbacterial effector protein secreted via the conjugative Vbh T4SS that is closely related to the VirB T4SS and encoded by plasmid pVbh of *B*. *schoenbuchensis*. We therefore propose that the Vbh T4SS together with its effector VbhT represent an evolutionary missing link on a path that leads from a regular conjugation system and FicTA toxin-antitoxin modules to the VirB T4SS and the Beps. Intriguingly, phylogenetic analyses revealed that the fusion of FIC and BID domains has probably occurred independently in VbhT and the common ancestor of the Beps, suggesting parallel evolutionary paths. Moreover, several other examples of TA module toxins that are *bona fide* substrates of conjugative T4SS indicate that their recruitment as interbacterial effectors is prevalent and serves yet unknown biological functions in the context of bacterial conjugation. We propose that the adaptation for interbacterial transfer favors the exaptation of FicT and other TA module toxins as inter-kingdom effectors and may thus constitute an important stepping stone in the evolution of host-targeted effector proteins.

## Author summary

Many bacterial pathogens use secretion systems to translocate effector proteins into host cells where they manipulate cell functions in favor of the pathogen. It is well-known that these secretion systems evolved from ancestors with functions in genuine bacterial contexts, but the origins of their secreted effectors have largely remained elusive. In this article we studied the evolutionary history of a host-targeting effector secretion system of the mammalian pathogen *Bartonella* that belongs to a group of machineries descended from secretion systems originally mediating DNA transfer between bacterial cells. Intriguingly, we found that such a DNA transfer machinery closely related to the host-targeting secretion system of *Bartonella* has recruited a bacterial protein involved in modulating DNA topology as an interbacterial effector protein that is translocated together with the DNA into recipient cells. The overall setup of this interbacterial effector is remarkably similar to the host-targeted effectors of *Bartonella*, and we propose that it represents an evolutionary missing link on the path from a genuine bacterial protein to effectors that manipulates host cell functioning. Further analyses showed that interbacterial effectors in DNA transfer may be a more common phenomenon and represent an important reservoir for the evolution of new host-targeted effectors.

## Introduction

The virulence of many bacterial pathogens depends on effector proteins that are translocated by dedicated secretion systems into eukaryotic host cells where they manipulate various processes in favor of the bacterium [1]. Previous work established that these host-targeting secretion systems have evolved from ancestors functioning in genuine bacterial contexts such as flagellation (type III secretion systems, T3SS), conjugation (type IV secretion systems, T4SS), or bacteriophage infection (type VI secretion systems (T6SS) [2–4]. However, the origins of effector proteins secreted by these diverse virulence factors have remained largely elusive, and their evolutionary histories are particularly difficult to trace due to the frequent horizontal gene transfer of functional effectors between bacterial pathogens [5–8].

One exception is the α-proteobacterial genus *Bartonella* that has evolved three distinct sets of effectors from a single common ancestor through independent series of gene duplication and diversification [9–11]. The *Bartonella* effector proteins (Beps) are translocated into host cells through the VirB T4SS of these ubiquitous mammalian pathogens and have been studied both for their functions in host cell subversion and for their roles in *Bartonella* evolution [12]. Host-targeting T4SS machineries have evolved various times independently from bacterial conjugation systems, i.e., from T4SS machineries that mediate the interbacterial transfer of plasmids [2, 13]. The VirB T4SS of *Bartonella* is the sister group of a putative conjugation system called VirB-homologous (Vbh) T4SS found on plasmids scattered over the genus *Bartonella* [10, 11, 14, 15]. Beyond *Bartonella*, the group formed by Vbh and VirB T4SS is closely related to conjugative T4SS machineries of other Rhizobiales such as the AvhB T4SS on pAT of *Agrobacterium tumefaciens* [11, 15]. Apart from the T4SS apparatus, conjugative plasmid transfer depends on a DNA processing and transfer (Dtr) machinery based on a protein called relaxase that is the actual substrate of the conjugative T4SS and gets covalently linked to the plasmid DNA prior to secretion. A type IV secretion coupling protein (T4CP) links Dtr and T4SS functionalities by mediating substrate selection for the T4SS machinery (recently reviewed by reference [13]).

In the most common and probably ancestral setup, the Beps secreted through the VirB T4SS of *Bartonella* have a characteristic bipartite domain architecture with an N-terminal FIC (filamentation induced by cAMP) domain and a C-terminal BID (Bep intracellular delivery) domain [9, 11]. While the BID domain forms the core of a type IV secretion signal shared with relaxases of related rhizobial conjugation systems [15–17], the FIC domain is an enzymatic domain that usually mediates the AMPylation of target proteins, i.e., the transfer of an adenosine 5’-monophosphate (AMP) [18]. We previously showed that the FIC domains of different Beps AMPylate distinct host proteins and are evolutionarily related to FicT toxins of the FicTA toxin-antitoxin (TA) module [19–21].

TA modules are small genetic elements consisting of a toxin protein that inhibits bacterial growth by interfering with essential cellular processes and a cognate antitoxin that protects the cell from the toxin, usually through direct protein-protein interaction [22]. The molecular activities of TA module toxins differ widely between RNases of the RelE, MazF, or VapC families, inhibitors of ribosomal translation like the Doc family, or enzymes that inactivate cell wall precursors like the PezT family [22–24]. We previously showed that FicT toxins AMPylate and concomitantly inactivate DNA gyrase and topoisomerase IV, two essential enzymes that control bacterial DNA topology [21]. Though TA modules are found abundantly in bacterial genomes, they are particularly enriched on mobile elements where they had originally been discovered as the mediators of post-segregational killing. This mode of “plasmid addiction” relies on the differential stability of TA module toxin and antitoxin in a way that, upon loss of the mobile element encoding a TA module, degradation of the antitoxin frees the toxin and causes cell death [25]. Post-segregational killing had initially been described as mechanism that enforces the stable vertical transmission of mobile elements, but more recent studies indicated that it may also be a weapon in the arms race of competing mobile elements: After horizontal gene transfer has brought together two incompatible elements in one cell, the TA module toxins encoded on only one element will kill the daughter cell receiving the other one after distributive segregation [26–28].

Interestingly, TA module toxins have multiple evolutionary links to host-targeted effectors of bacterial pathogens that are by far not restricted to the FicT-like FIC domains of Beps. For example, structural and bioinformatic work showed that the core of AvrB, a T3SS effector of the plant pathogen *Pseudomonas syringae*, is homologous to the Doc toxin of the Phd/Doc TA module [29]. Similarly, a recent study on the plant pathogen *Xanthomonas oryzae* pv. *oryzicola* showed that AvrRxo1, another T3SS effector, is homologous to the PezT toxin of the PezTA toxin-antitoxin module [30]. The Doc and PezT toxins are kinases that phosphorylate and thereby inactivate the translation elongation factor Tu or the peptidoglycan precursor UDP-N-acetylglucosamine (UNAG), respectively [23, 24]. It appears that evolution has shaped these enzymatic machineries to mediate the phosphorylation of nicotinamide adenine dinucleotide (NAD) in case of AvrRxo1 or, for AvrB, to give up own enzymatic activities and interfere with plant immunity through protein-protein interactions [31–33]. However, the evolutionary trajectories of how host-targeted effectors can arise from TA module toxins have remained largely elusive.

In this study, we show that the Vbh T4SS encoded on pVbh of *Bartonella schoenbuchensis* functions as a classical conjugation system that is genetically and evolutionarily linked to the FicTA-family toxin-antitoxin module VbhTA. Like the Beps, the VbhT toxin is a composite protein formed by the fusion of a FicT-like FIC domain and a relaxase-derived BID domain. Intriguingly, we demonstrate that VbhT is an interbacterial effector protein translocated alongside conjugative plasmid transfer of pVbh into recipient cells. Targeted sequence analyses revealed several additional, independent recruitments of TA module toxins as *bona fide* interbacterial effector proteins in the context of conjugative plasmid transfer. We therefore propose that the recurrent acquisition of TA module toxins as interbacterial effectors may be an important evolutionary missing link that facilitates the exaptation of genuine bacterial proteins as host-targeted virulence factors.

## Results

### BtrFicTA is a typical FicTA module encoded in the remnants of a conjugative plasmid in *Bartonella*

**Fig 1:**
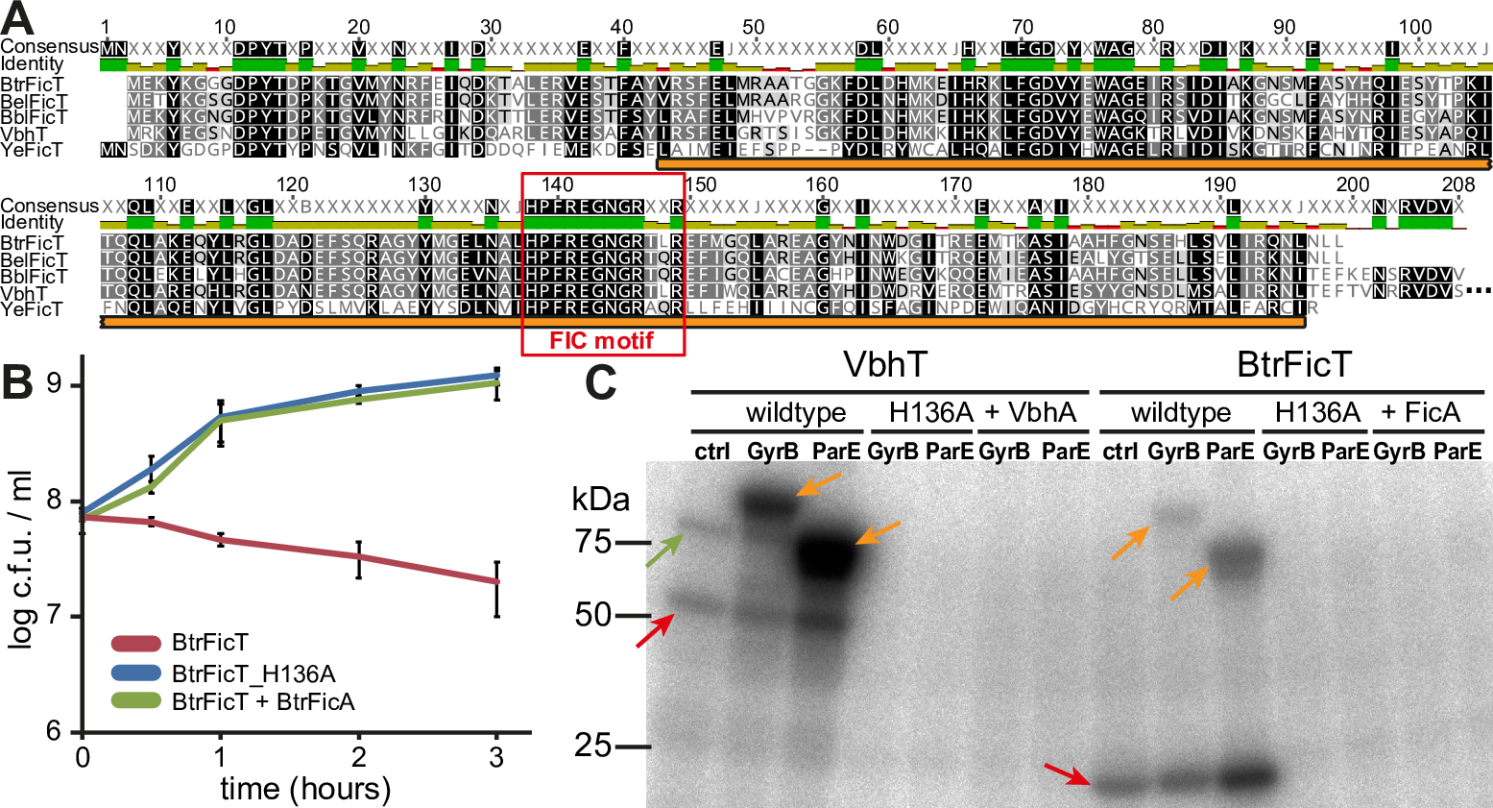
BtrFicTA is a regular FicTA module closely related to VbhTA. (A) Sequence alignment of YeFicT (UniProt identifier A1JNF3 (A1JNF3_YERE8)) and the FIC domain of VbhT (UniProt identifier E6Z0R3 (VBHT_BARSR)) with BtrFicT (UniParc identifier UPI00015FA8A2) and orthologs encoded by *Bartonella elizabethae* (BelFicT; UniParc identifier UPI00026E5C06) and *Bartonella birtlesii* (BbiFicT; UniParc identifier UPI00026E6E87). The FIC domain core (interpro IPR003812) is highlighted with an orange bar. BtrFicT and its orthologs have > 70% identical sequence and share 60% sequence identity with the FIC domain of VbhT. The four proteins have 28% identical sequence with YeFicT. Note that all sequences display a canonical HPFX[D/E]GNGRXXR FIC signature motif (red frame), indicating AMPylation as their molecular activity [34]. The coloring indicates amino acid similarity according to the Blosum62 score matrix with black = 100% identity and white = <60% identity. (B) The colony forming units (c.f.u.) / ml of exponentially growing *E*. *coli* were recorded over time after the expression of BtrFicT constructs had been induced at t = 0 h with 2 mM of IPTG. Data points represent average and standard deviation of three independent experiments. (C) Autoradiograph of an AMPylation assay with lysates of *E*. *coli* that had expressed different VbhT or BtrFicT constructs. Reactions were set up by adding [α-^32^P]-ATP to trace AMPylation and lysates of *E*. *coli* that expressed GST-fusions of *E*. *coli* GyrB (DNA gyrase B subunit), *E*. *coli* ParE (topo IV B subunit), or a vector control. The autoradiograph shows VbhT and BtrFicT auto-AMPylation (red arrows), AMPylation of endogenous *E*. *coli* GyrB by VbhT (green arrow), and the AMPylation of ectopically expressed GST-GyrB and GST-ParE for both constructs (orange arrows). Note that the “BtrFicT” construct included expression of a marginal amount of BtrFicA which we determined to be necessary for the generation of soluble BtrFicT (see S1 Text).

**Fig 2:**
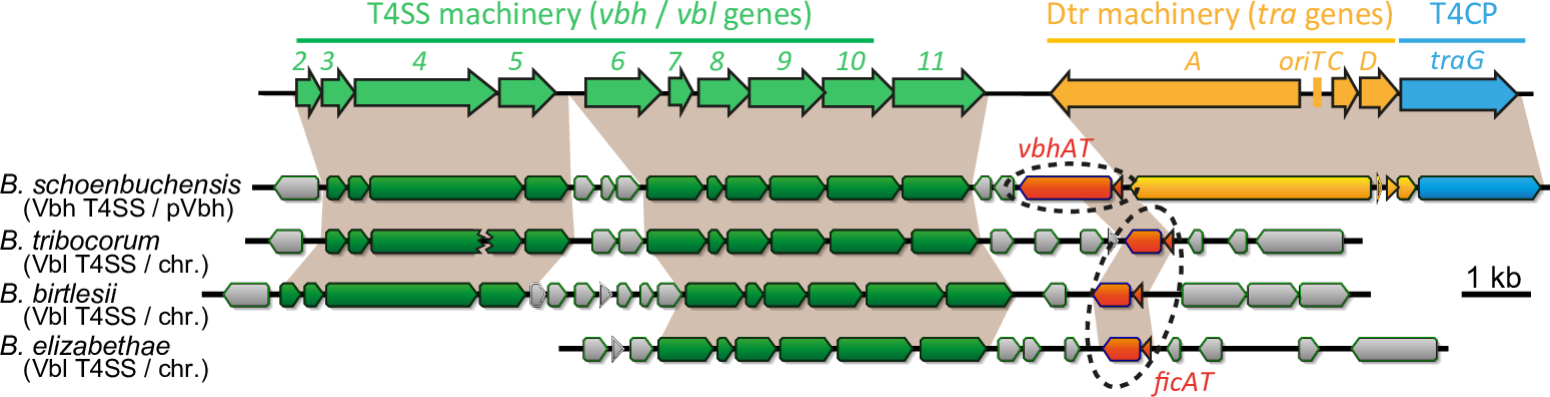
BtrFicTA and related FicTA modules are encoded with deteriorated conjugative T4SS machineries. Loci encoding bacterial conjugation systems consist of genes for the T4SS machinery (green), the Dtr machinery (yellow), and a type IV coupling protein (T4CP) that links these functions by recruiting the Dtr machinery to the T4SS (blue). Like VbhTA on pVbh, BtrFicTA of *B*. *tribocorum* and its orthologs are encoded directly downstream of the T4SS machineries (annotated as Vbl T4SS for “VirB-like”).

In our previous work we studied the molecular activities of the VbhTA module of *B*. *schoenbuchensis* that differs from all other characterized FicTA modules in that the VbhT toxin contains a C-terminal BID domain, the hallmark of *Bartonella* type IV secretion substrates [21]. Interestingly, several other bartonellae encode FicTA modules that are closely related to VbhTA but lack a BID domain at the C-terminus of their FicT family toxins (Fig 1A). We examined one of these, BtrFicTA of *Bartonella tribocorum* str. CIP105476, more closely and found that–similar to VbhT or other FicT toxins–ectopically expressed BtrFicT inhibited the growth of *E*. *coli* unless expression of its cognate antitoxin BtrFicA was induced as well (Fig 1B). Like VbhT, BtrFicT AMPylated DNA gyrase and topo IV of *E*. *coli* in *in vitro* assays, and the growth inhibition caused by BtrFicT in *E*. *coli* depended on the catalytic activity of the BtrFicT FIC domain and absence of the BtrFicA antitoxin (Fig 1B and 1C). It therefore appears that BtrFicTA is a classical FicTA-family TA module similar to YeFicTA of *Yersinia enterocolitica* or PaFicTA *Pseudomonas aeruginosa*, two FicTA modules that we had studied previously [21]. Interestingly, BtrFicTA and orthologous FicTA modules in other bartonellae are encoded in genomic islands related to plasmids like pVbh of *B*. *schoenbuchensis* or pBGR3 of *Bartonella grahamii* that are prevalent in some lineages of *Bartonella* [11, 14] (Fig 2). Similar to these plasmids, the genomic islands invariably encode a Vbh T4SS, but the chromosomal machineries show different signs of deterioration like pseudogenization of key components and consistently lack Dtr functions as well as a T4CP [10, 14] (Fig 2).

### pVbh of *Bartonella* is a conjugative plasmid encoding numerous TA modules

**Fig 3:**
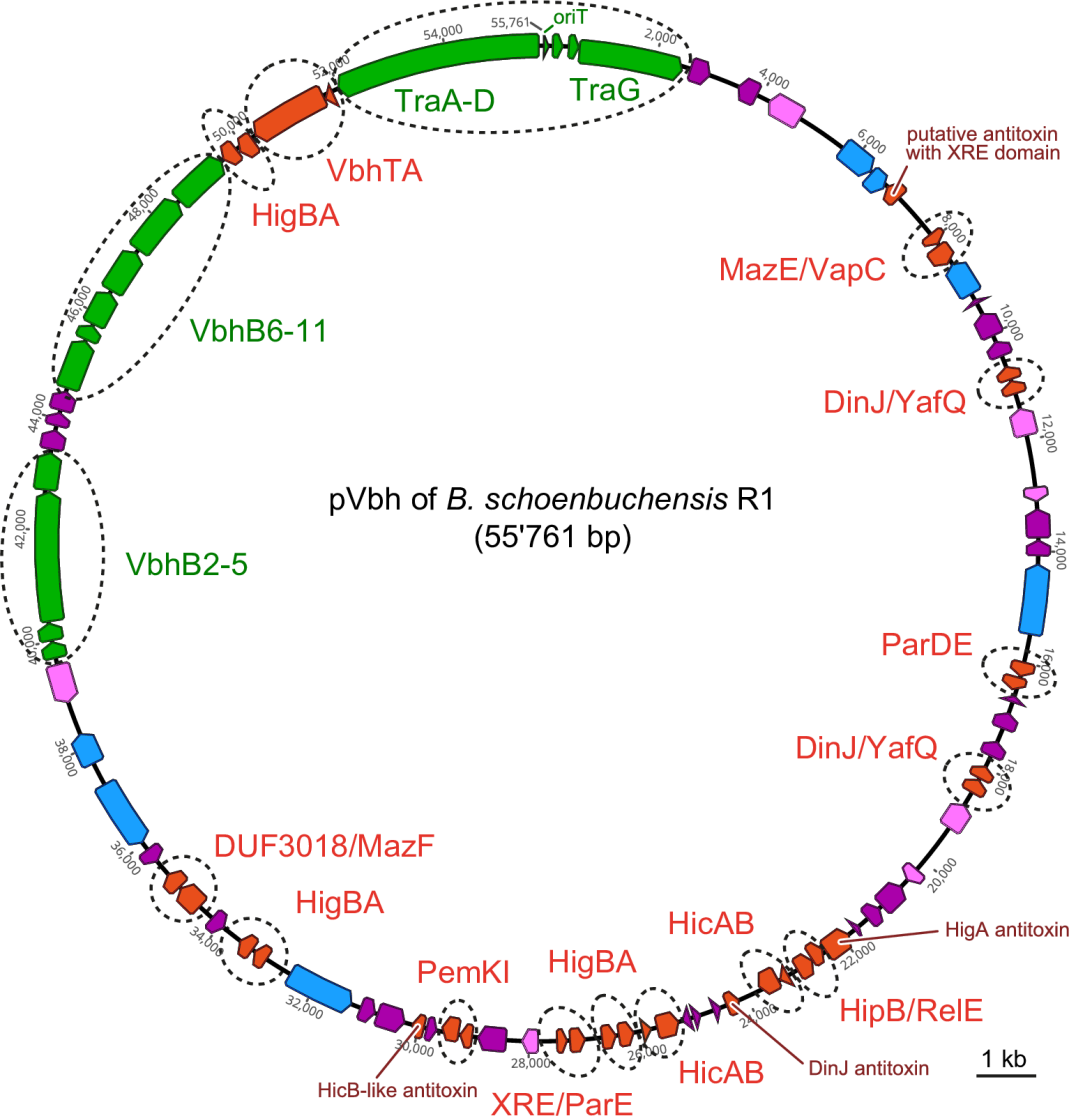
pVbh encodes a multitude of TA modules but lacks any recognizable cargo genes. All genes on pVbh of *B*. *schoenbuchensis* R1 (genbank accession number CP019790.1) were manually annotated and categorized as belonging to the Vbh conjugation system (green; see also Fig 2), plasmid replication and partitioning functions (light blue), TA modules (red), or as encoding proteins of unknown function with (light pink) or without (dark pink) known protein domains. The fourteen predicted TA modules and four orphan antitoxins as well as the loci encoding the Vbh T4SS (VbhB2-11), Dtr functions (TraA-D), and T4CP (TraG) were highlighted in detail. Annotations of every gene on pVbh are available in S1 Fig.

The close relationship of Bep FIC domains and FicT toxins on one side and the Vbh and VirB T4SS machineries on the other side suggested that the pVbh and Vbh T4SS may have features that are informative regarding the conjugative ancestors of the VirB T4SS and their evolution into host-targeting machineries. We therefore manually annotated all genes on pVbh of *B*. *schoenbuchensis* R1 in order to get a basic insight into the biology of this *bona fide* conjugative replicon (Fig 3 and S1 Fig). Apart from plasmid replication functions and a complete conjugation machinery including the Vbh T4SS, pVbh apparently only encodes a number of hypothetical genes and a substantial arsenal of fourteen TA modules (Fig 3). Some toxin-antitoxin pairings are non-canonical like, e.g., a TA module formed by a MazE family antitoxin and a VapC family toxin (known from the MazEF and VapBC modules, respectively [22]). However, it is known that the association of TA module toxins and antitoxins is highly variable in a way that toxins of a given family can often form functional TA modules with several different antitoxin families and vice versa (see, e.g., the analysis of Leplae et al. [35]). In addition to complete TA modules, pVbh encodes four orphan antitoxins that may have “anti-addiction” functions by interfering with post-segregational killing based on TA modules with cognate toxins on other replicons [36]. pVbh seems to lack any classical cargo genes like metabolic operons or virulence factors, suggesting that this plasmid is a mobile parasite that relies on TA modules to overpower its host and competing replicons (Fig 3 and S1 Fig).

**Fig 4:**
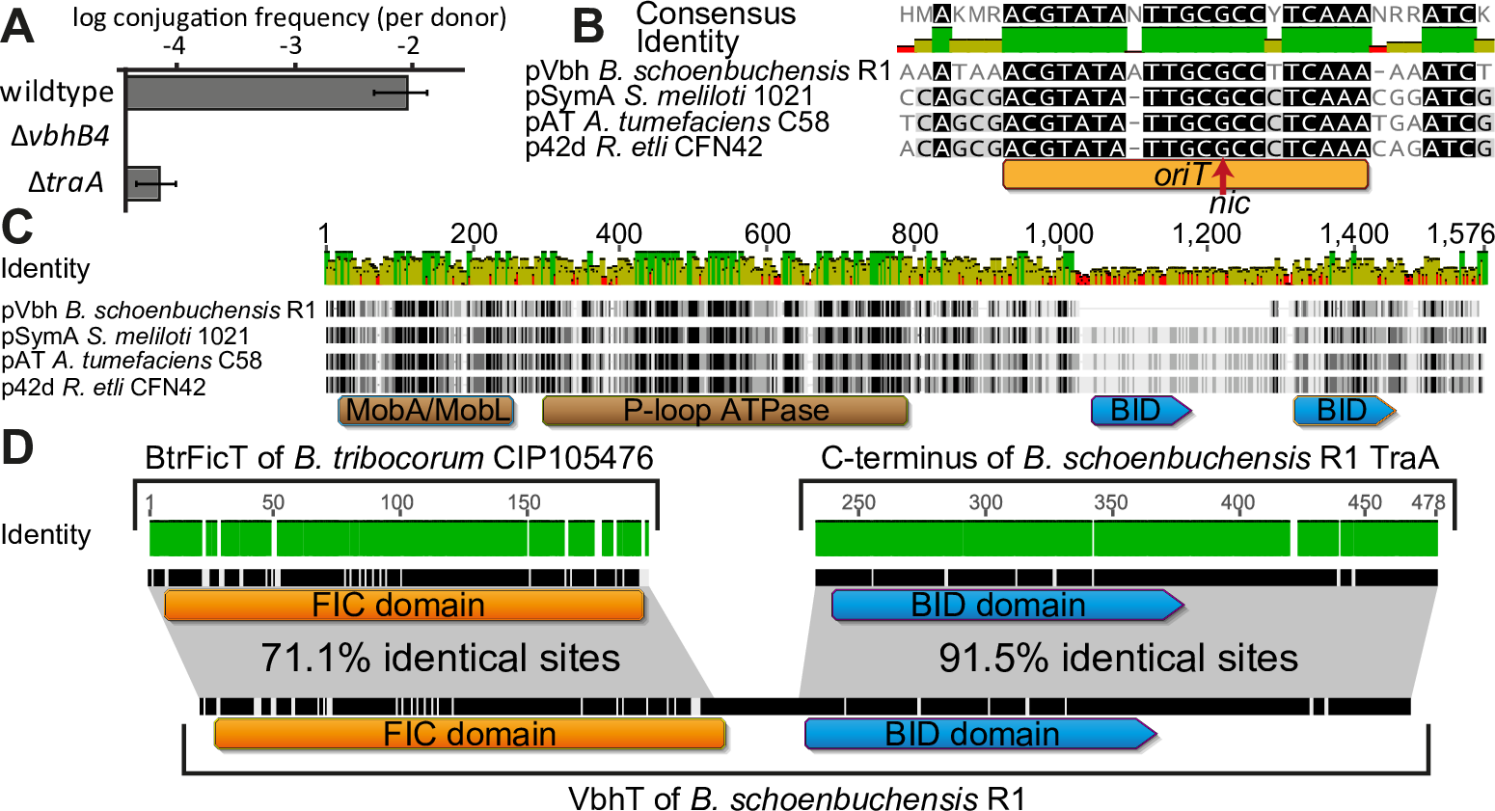
pVbh as a conjugative replicon with composite toxin VbhT. (A) Filter matings of *B*. *schoenbuchensis* R1 donors and *B*. *henselae* recipients revealed that pVbh conjugates at a frequency of ca. 1% per donor. Conjugative transfer depended on a functional Vbh T4SS (*vbhB4* mutant) and its Dtr functions (*traA* mutant). Data points and error bars represent mean and standard deviation of at least three independent experiments. (B) The origin of conjugative DNA transfer (*oriT*, orange bar) on pVbh was inferred by comparison to closely related rhizobial plasmids where this sequence and the actual site of relaxase cleavage (*nic*) had been experimentally determined [16]. All these plasmids invariably encode *oriT* between the *traA* relaxase and the *traCD* relaxasome components in the *dtr* region (see Fig 2). (C) Domain composition and sequence alignment of the TraA relaxase of pVbh with relaxases of closely related rhizobial conjugation systems. (D) The alignment of BtrFicT, VbhT, and TraA protein sequences shows that VbhT is a composite protein with an N-terminal FIC domain closely related to FicT toxins of *Bartonella* and a C-terminal secretion signal virtually identical to homologous sequence of the TraA relaxase.

Matings of *B*. *schoenbuchensis* R1 and the *B*. *henselae* Houston-1 strain revealed interbacterial transfer of pVbh at a frequency of around 1/100 per donor (Fig 4A). This result is well in the range of transfer frequencies observed with other natural conjugative plasmids including, e.g., pAT of *A*. *tumefaciens* that carries the AvhB conjugation system closely related to the Vbh machinery [37–39]. As expected, conjugation of pVbh depended on the Vbh T4SS and its Dtr functions (Fig 4A). Furthermore, we inferred the origin of transfer (*oriT*) of pVbh in the *dtr* region by comparison to the closely related conjugation systems of other Rhizobiales (Fig 4B) and found considerable similarities also among their relaxases, with the interesting exception that the relaxase of pVbh has only one and not two BID domains like its close relatives (Fig 4C). Similar to genomic islands harboring BtrFicTA and its orthologs, pVbh encodes the VbhTA module downstream of the *vbh* machinery and upstream of the *dtr* genes (absent in the chromosomal loci; Fig 2). Conspicuously, the BID domain of the VbhT toxin is virtually identical to the BID domain of the TraA relaxase of pVbh (Fig 4D). We therefore hypothesized that the VbhT toxin may be secreted between bacterial cells, unlike classical FicT toxins that likely have functions inside bacterial cells like other TA module toxins.

### A new CRAfT assay enables the detection of interbacterial protein transfer with high specificity and sensitivity

**Fig 5:**
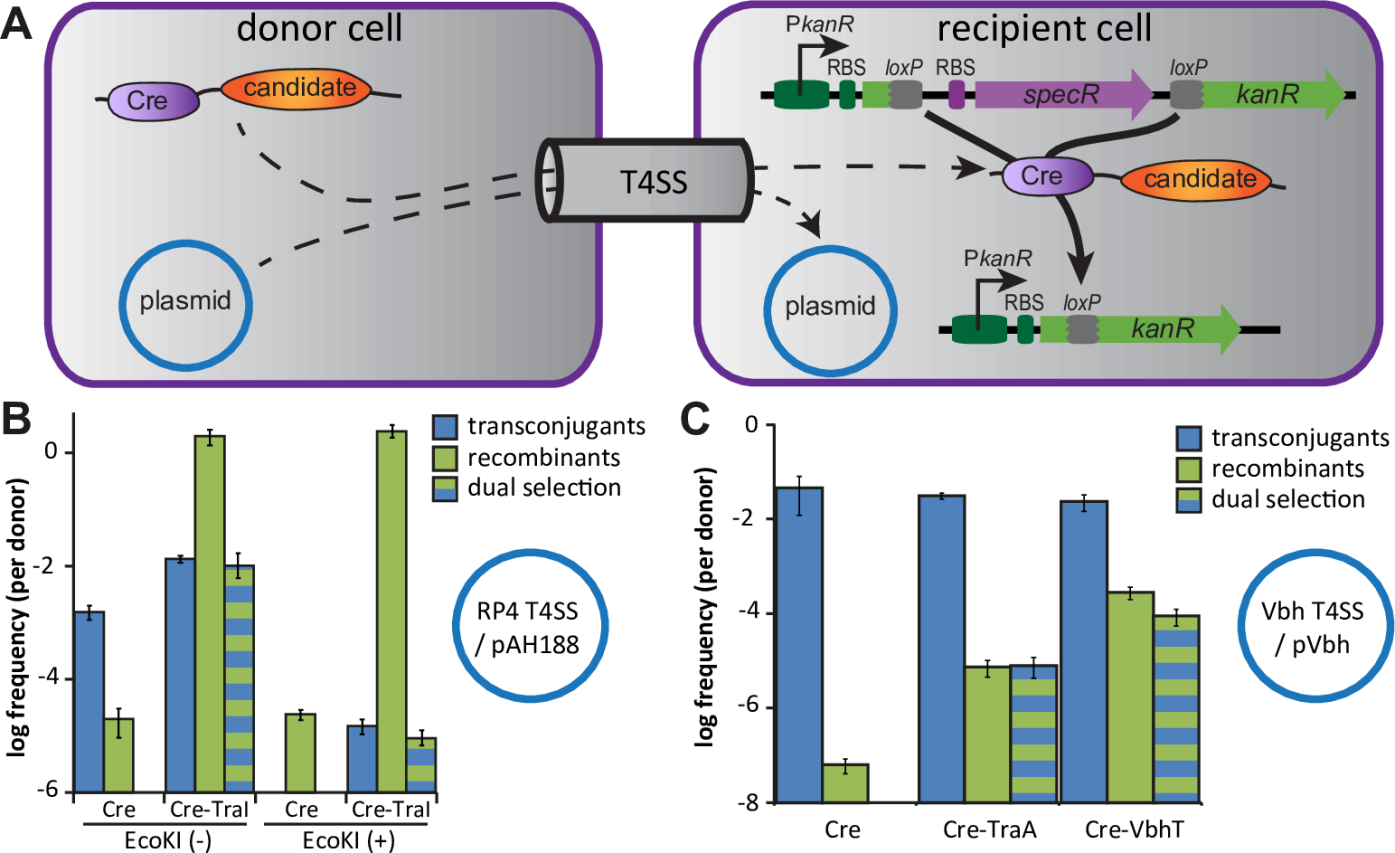
A novel variant of CRAfT detects the interbacterial transfer of VbhT. (A) The scheme outlines basic principles of the new CRAfT variant that we developed as part of this study. Interbacterial protein transfer of Cre fusions is detected through the switch from spectinomycin resistance (purple) to kanamycin resistance (green). Conjugative plasmid transfer can be assayed in parallel (blue) by selection for antibiotic resistance encoded on reporter plasmid pAH188 (chloramphenicol resistance; *E*. *coli* matings) or pVbh (gentamicin resistance; *Bartonella* matings). (B) Conjugative transfer of pAH188 and CRAfT signal for the translocation of a Cre-TraI relaxase fusion through the RP4 T4SS were assayed using *E*. *coli* K-12 BW25113 (lacking EcoKI) or *E*. *coli* K-12 MG1655 (with functional EcoKI) as recipients. (C) The transfer of Cre fused to the TraA relaxase of pVbh or the catalytically inactive H136A mutant of VbhT were tested in matings of *B*. *schoenbuchensis* with *B*. *henselae* carrying the CRAfT sensor module; conjugation of pVbh was assayed in parallel. Data points and error bars in (B) and (C) represent mean and standard deviation of three independent experiments. Transconjugants are recipient cells that have received pAH188 (B) or pVbh (C), and recombinants denote recipient cells that switched resistance of the CRAfT sensor due to successful transfer of Cre fusion proteins or, much rarer, spontaneous recombination of the *loxP* sites.

CRAfT (Cre recombinase assay for translocation) is a genetic approach to detect intercellular protein secretion. For CRAfT, candidate proteins are expressed in donor cells as translational fusions to the Cre site-specific recombinase of bacteriophage P1 and the recipient cells encode a genetic module that detects translocation of Cre through activation of marker genes upon recombination of its *loxP* target sites [40]. Though typically used to detect the translocation of bacterial effectors into eukaryotic host cells [15, 40, 41], CRAfT has previously also been employed for the detection and quantification of interbacterial protein transfer in the context of conjugation, i.e., usually the translocation of relaxase proteins [42, 43]. Since the genetic features of these assays made them difficult to use in *Bartonella*, we constructed a new variant of CRAfT that was designed to be easily applicable in many different organisms (see S1 Text). In short, we placed the CRAfT recipient sensor module on a Himar1 transposon and designed it to detect translocation of Cre fusion proteins as a switch from spectinomycin to kanamycin resistance (Fig 5A). This setup uses antibiotics enabling efficient selection in many bacteria and allows selection against spontaneous *loxP* recombination prior to the actual experiment. The donor plasmid for the expression of Cre fusions was constructed based on the new, versatile broad host-range expression plasmid pBZ485 that we also constructed as part of this study (S1 Text).

For a proof of concept, we used the well-characterized conjugative RP4 T4SS encoded in the chromosome of *E*. *coli* donor strain JKE201 (see S1 Text). Matings with *E*. *coli* K-12 recipients carrying the CRAfT sensor module revealed that expression of Cre fused to the TraI relaxase of RP4 in the donor resulted in *loxP* recombination at a frequency of ca. 100% per donor cell (Fig 5B). Conversely, parallel conjugative transfer of reporter plasmid pAH188 with a minimal RP4 *oriT* was far less efficient under our experimental conditions (0.1%-1% per donor; Fig 5B), enabling us to study the link between plasmid and protein transfer by dual selection. The pAH188 transconjugants were all among the cells that had received Cre-TraI (i.e., the recombinants) despite a large excess of recipients (Fig 5B). As expected from the role of TraI as the conjugative relaxase, this result demonstrates that conjugative DNA transfer and the detection of protein translocation by our new variant of CRAfT are linked (Fig 5B). We then switched the recipient from *E*. *coli* K-12 BW25113 to the MG1655 wildtype strain that encodes a functional EcoKI restriction-modification system. Since pAH188 harbors three EcoKI sites and the *ecoKI* locus had been deleted during the construction of our *E*. *coli* JKE201 donor strain (see S1 Text), the switch of recipients greatly reduced conjugative transfer of this plasmid (Fig 5B). However, the CRAfT signal was not affected by the change of recipient, confirming that our CRAfT assay detected true protein transfer through the RP4 T4SS (Fig 5B). Similarly, our new variant of CRAfT could also detect the translocation of Cre-relaxase fusions through the very distinct conjugative machinery of the *E*. *coli* F-plasmid (S2 Fig).

### VbhT is secreted via the Vbh T4SS alongside plasmid transfer

In order to assess whether our new variant of CRAfT was also suitable to assay protein transfer through the Vbh T4SS of *Bartonella*, we created a Cre-fusion of the conjugative relaxase TraA of pVbh and assayed its translocation through the Vbh T4SS during bacterial conjugation from *B*. *schoenbuchensis* into *B*. *henselae*. As expected, TraA was found to be translocated into recipient cells alongside conjugative transfer of pVbh, although the sensitivity of CRAfT seemed to be lower in *Bartonella* than in *E*. *coli* (Fig 5C). Furthermore, we could clearly show that a Cre-VbhT fusion construct is secreted from *B*. *schoenbuchensis* into *B*. *henselae* during conjugative transfer of the pVbh plasmid (Fig 5C; using a non-toxic mutant of VbhT). Carrying the same molecular activities as other FicT toxins [21], VbhT therefore seems to double as an interbacterial effector that is secreted during conjugation into recipient cells.

### Repeated evolution of toxin transfer alongside bacterial conjugation

**Fig 6:**
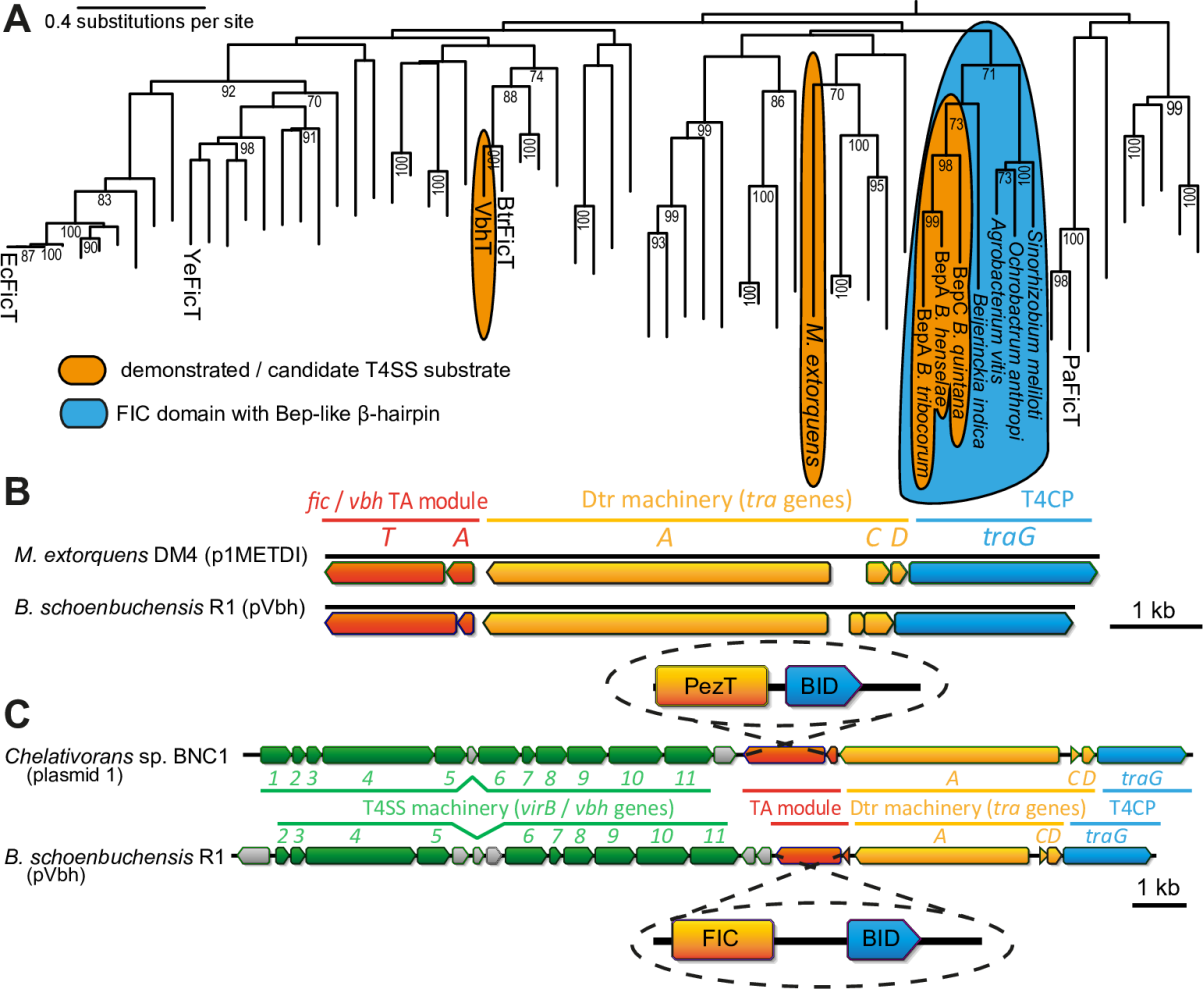
TA module toxins as *bona fide* substrates for conjugative type IV secretion. (A) The position of known and proposed T4SS substrates as well as proteins with a Bep-like β-hairpin are highlighted in the phylogeny of FicT toxins (adapted from our previous work [21]; see also S3A–S3C Fig). In short, the tree represents a maximum likelihood phylogeny that had been constructed from an alignment of the toxins’ FIC domains. An additional phylogeny supporting the repeated, independent recruitment of FicT toxins as T4SS substrates is presented in S3B Fig. FicT of *M*. *extorquens* (UniProt identifier C7CN81 (C7CN81_METED)) is shown as a candidate T4SS substrate because it carries a BID-like sequence at its C-terminus (S3C Fig). (B) The genetic arrangement of *vbhAT* at the Dtr locus of pVbh of *B*. *schoenbuchensis* R1 was compared to the locus encoding the FicTA module on p1METDI of *M*. *extorquens* DM4 (genbank accession number NC_012987). (C) The illustration shows how a PezTA module on *Chelativorans* sp. BNC1 plasmid 1 (genbank accession number CP000389.1) is encoded between the conjugative VirB T4SS and its Dtr machinery in the same genetic arrangement as the VbhTA module is encoded on pVbh.

The phylogeny of FIC domains shows that the Beps and VbhT are each most closely related to FicT toxins without C-terminal BID domains (Fig 6A). Furthermore, the Beps and a small group of closely related FicT toxins, but not VbhT or any other FIC domain proteins, carry a short β-hairpin of unknown function that strongly suggests common ancestry (Figs 6A and S3A) [11]. It is therefore apparent that the Beps and VbhT acquired their BID domains through independent domain fusion events and represent two paths of parallel evolution. Consequently, the interbacterial effector VbhT is not ancestrally related to the Beps, but likely still represents an ancestral state in the evolution of the Beps prior to their adaptation for inter-kingdom secretion. We also discovered additional examples for the independent evolution of TA module toxins into *bonda fide* T4SS substrates. Interestingly, the genetic arrangement of VbhTA and the Vbh T4SS on pVbh was exactly mirrored by a FicTA module encoded with a seemingly conjugative T4SS on plasmid p1METDI of *Methylobacterium extorquens* DM4. Just like in *Bartonella*, the TA module on the *M*. *extorquens* plasmid is encoded directly downstream of the relaxase in proximity to the other *dtr* genes and has a C-terminal extension beyond the FIC domain of the toxin that aligns to BID domains (Figs 6B and S3C). Despite these similarities, the FIC domain of this *M*. *extorquens* FicT toxin is only very distantly related to the FIC domains of VbhT or the Beps and seems to have acquired its C-terminal BID-like extension in a third, separate domain fusion event (Fig 6A). Consistently, the relaxases of pVbh and p1METDI are only distantly related (S3D Fig). In addition to the Beps, VbhT, and FicT of *M*. *extorquens*, others have previously proposed that a distinct group of FIC domain proteins encoded with an apparently conjugative T4SS in *Campylobacter* may serve as interbacterial effectors, though this remains to be demonstrated experimentally [43]. Beyond FIC domain proteins, we found another interesting TA module is encoded on plasmid 1 of *Chelativorans* sp. BNC1, inhabitants of the phyllosphere that are closely related to *Mesorhizobium*. In this case, the toxin of a PezTA module harbors a C-terminal BID domain and is found in a genetic arrangement strikingly similar to VbhTA and the Vbh T4SS on pVbh, suggesting that this toxin is a substrate of the conjugative T4SS encoded next to it (Fig 6C). Taken together, these results suggest that TA module toxins of different families have repeatedly evolved into substrates of interbacterial type IV secretion alongside conjugative plasmid transfer.

## Discussion

**Fig 7:**
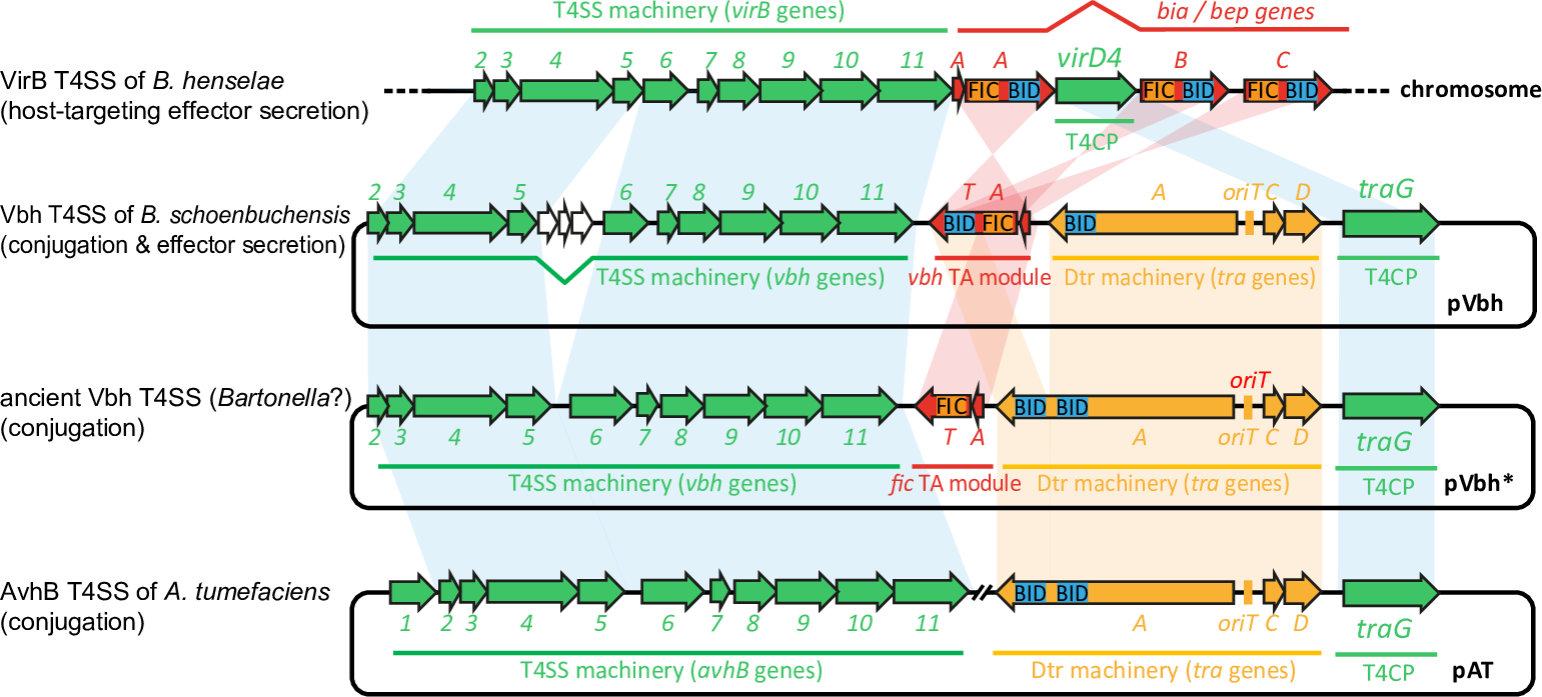
Working model: A VbhT-like interbacterial effector as a missing link in the evolution of Beps from FicT toxins. The model illustrates the homology of genes associated with different T4SS machineries ranging from a regular conjugation system (represented by the AvhB T4SS of *A*. *tumefaciens*, bottom) to a host-targeting, effector secreting virulence factor (VirB T4SS of *B*. *henselae*, top). Core components of the T4SS machinery and the T4CP are shown in green, the Dtr machinery is shown in yellow, and TA modules or effectors are shown in red. Note that the VbhTA module of *B*. *schoenbuchensis* pVbh (second from top) displays the same domain architecture as the majority of extant Beps and likely their common ancestor. Like VbhT, the most upstream Bep (BepA) is encoded with a FicA antitoxin that is called BiaA in the context host-targeted *Bartonella* effectors [11]. Based on the genomic islands with Vbh-like T4SS and FicTA modules like BtrFicTA (Fig 4), we infer an ancient pVbh-like plasmid in which a FicTA module was encoded between the conjugative relaxase and the T4SS machinery (second from bottom). It is clearly apparent that a DNA rearrangement fusing one BID domain of the relaxase with the FicT toxin–i.e., classical terminal reassortment–would create a VbhT-like interbacterial effector as evidenced by the clear composite architecture of this protein (Fig 6). We therefore speculate based on these homologies that an evolutionary process from the bottom to the top of this model may approximate the evolutionary history of the host-targeting VirB T4SS.

While it is easily conceivable how interbacterial secretion systems like conjugation machineries may have been repurposed to simply target a different cell type for their exaptation as virulence factors, the deep evolutionary origins of translocated effectors have remained elusive. In the most basic setup, effector proteins like the Beps comprise two functional modules of which one is used to subvert host cell functions and the other one serves as a translocation signal for the secretion system. This modular architecture facilitates the formation of effectors through random genetic fusions of a suitable secretion signal with any functional sequence stretch, a process that has been called “terminal reassortment” [44]. Such a model of quantum leap evolution is well-suited to explain the wide variety of different effectors that can be secreted through a single machinery like the Dot/Icm T4SS of *Legionella* and is in accord with the occasional direct recruitment of host-derived sequences for effector formation [6]. However, it does not tell about the actual bacterial genes that had been recruited for the formation of extant host-targeted effectors or the evolutionary processes that shaped their conversion from genuine bacterial factors into secreted tools to subvert host cell signaling. In this study we showed that the recruitment of TA module toxins as interbacterial effectors may be a stepping stone on this evolutionary path. The composite domain architecture of VbhT is a clear “smoking gun” that reveals the *de novo* evolution of an interbacterial effector through the fusion of a FicT toxin with the BID domain of a conjugative relaxase (Fig 4D). The close genetic association of BtrFicTA-like TA modules and the Vbh T4SS on an ancestral pVbh-like plasmid may have favored such a DNA rearrangement (Figs 2 and 7), and this event may also explain why the TraA relaxase of pVbh carries only one BID domain and not two like its close homologs (Figs 4C and 7). Interbacterial effectors as evolutionary missing links uncouple the exaptation of bacterial proteins for intercellular transfer (through terminal reassortment) from their adaptation for host interaction, so that the association with interbacterial secretion systems can be seen as a pre-adaptation for the function as a host-targeted effector. Given the close relationship of Vbh and VirB T4SS machineries of *Bartonella* and that VbhT displays the same domain architecture as the common ancestor of all Beps, we hypothesize that a primordial Bep has evolved from a VbhT-like protein in parallel to the exaptation of an ancestral, Vbh-like T4SS machinery for host interaction (Fig 7). However, our results show that the extant VbhT protein itself is not a “living fossil” ancestrally related to the Beps, but instead only one of several examples for the parallel evolution of FicT proteins and other TA module toxins into interbacterial effectors associated with conjugation systems. Beyond VbhT and the Beps, we found a possible third instance of a FicT toxin secreted through a conjugative T4SS on a plasmid of *M*. *extorquens* and a PezT toxin that may be the substrate of a conjugation system of *Chelativorans* sp. BNC1 (Fig 6 and S3 Fig). These proteins were identified as *bona fide* T4SS substrates based on a BID-like sequence at their C-terminus, but most T4SS machineries have only very short secretion signals based on a positively charged C-terminal region and are notoriously difficult to predict [15, 41, 42]. We therefore anticipate that future work will reveal additional TA module toxins that are secreted alongside conjugative DNA transfer.

The recruitment of host-targeted effectors from genuine bacterial ancestors via interbacterial effectors as a stepping stone like in case of FicT / Beps and likely also PezT / AvrRxo1 (Figs 6 and 7 and S3 Fig) is only one possible evolutionary path. Other effectors have likely been recruited directly via processes like terminal reassortment from bacterial genes or from host genes that have been acquired by horizontal gene transfer [6, 44], though their evolutionary history is often unclear. As an example, the *Legionella* effector DrrA AMPylates its host target Rab1b using a nucleotidyl transferase domain closely related to bacterial glutamine synthetase adenylyl transferase, an enzyme that regulates glutamine synthetase by AMPylation, suggesting that this domain may have been recruited from a bacterial housekeeping protein [45]. It would be interesting to see future studies unraveling and comparing the history of host-targeted effectors with different evolutionary background.

Though the evolutionary paths from conjugative to host-interacting T4SS machineries have not been studied in detail, it is tempting to speculate that this transition may have either happened directly or through an intermediate step in which an essentially conjugative T4SS machinery manipulates host cell surface structures without effector secretion. Examples for such an interaction are the Trw T4SS of *Bartonella* (mediating adhesion to red blood cells) or the Cag T4SS of *Helicobacter pylori* (manipulating integrins on the host cell surface) [46, 47]. In this context, it would also be interesting to study whether the chromosomal T4SS machineries closely related to the Vbh T4SS are merely vestiges of conjugation systems (as indicated by their lack of Dtr functions, T4CP, and any obvious substrates; Fig 2) or have a biological function. We speculate that these loci may promote *Bartonella* biofilm formation, because the membrane-spanning core machineries of several bacterial conjugation systems have been shown to induce biofilm formation of *E*. *coli* independent of actual substrate translocation [48]. *Bartonella* is particularly well-suited to unravel the evolution of host-targeted secretion systems and their effectors, because representatives of putative more ancestral evolutionary states are still around in the genus and can be directly studied like BtrFicT or the Vbh T4SS and VbhTA.

In this study we showed that the FicT toxin VbhT is secreted during bacterial conjugation as an interbacterial effector protein (Fig 5). These results were immediately reminiscent of previous work on the secretion of DNA primase alongside conjugative DNA transfer that seems to have evolved several times independently in order to promote plasmid replication in the recipient cell under unfavorable conditions [49–51]. Similarly, *A*. *tumefaciens* secretes a number of proteins together with the T-DNA that protect it and promote integration into the host cell genome [52]. It is therefore well imaginable that the secretion of FicT toxins as interbacterial effectors may also have evolved as an accessory function in conjugative plasmid transfer. VbhT seems to have the same molecular activities as regular FicT toxins, and their ability to globally modulate cellular DNA processing makes it plausible that they somehow support intercellular DNA transfer [21]. Alternatively, our finding that a PezT toxin may be secreted analogously to VbhT could suggest a more generic role for toxin secretion during bacterial conjugation, possibly as an advanced form of post-segregational killing. We note that the direct secretion of toxin proteins alongside conjugative plasmid transfer would initiate the addiction of recipient cells to the incoming plasmid already during the conjugative plasmid transfer. This form of plasmid addiction would be independent from the expression of plasmid-encoded genes in the recipient cells, so that defense mechanisms like restriction-modification systems could be overcome. Similarly, the secretion of plasmid-encoded bacteriocins can also eliminate plasmid-less cells and cause post-segregational killing, though independent of bacterial conjugation [53]. It has also been shown that a chromosomal T4SS of *Xanthomonas* secretes toxin proteins into neighboring cells with the aim to kill niche competitors in a way similar to interbacterial type V or type VI secretion [54–56]. Such a function seems unlikely for VbhT and other TA module toxins linked to conjugation systems, because their strong genetic and evolutionary association with the conjugative replicons is difficult to reconcile with the elimination of potential recipient cells.

## Materials and methods

### Bacterial handling

*Escherichia coli* was cultured using LB liquid or solid medium at 37°C. Media were supplemented with 1% w/v D-glucose to reduce basal expression of P*tac-lac* / P*lac* and P*ara* promoters in overnight cultures through catabolite repression whenever suitable. Selection for relevant genotypes or plasmid maintenance was performed with ampicillin ad 30 μg/ml (mini-R1 origin of replication) or 100 μg/ml (other origins of replication), chloramphenicol ad 34 μg/ml, gentamicin ad 20 μg/ml, kanamycin ad 50 μg/ml, spectinomycin ad 50 μg/ml, or zeocin ad 50 μg/ml. P*tac-lac* / P*lac* was induced with different concentrations of isopropyl β-D-thiogalactopyranoside (IPTG) and P*ara* was induced with 0.2% w/v of L-arabinose. All media for the growth of conjugation donor JKE201 and related strains were supplemented with 1,6-diaminopimelic acid (DAP) ad 1 mM to complement their auxotrophy.

*Bartonella* strains were grown on nutrient agar plates supplemented with 5% defibrinated sheep blood in a humidified atmosphere with 5% CO_2_ at 35°C for 3–7 days. *B*. *henselae* Houston-1 was cultured on Columbia agar or heart-infusion agar, while *B*. *schoenbuchensis* R1 was grown on tryptic soy agar or heart-infusion agar (all from Oxoid). Plasmid maintenance was selected with gentamicin ad 10 μg/ml, kanamycin ad 30 μg/ml, or spectinomycin ad 50 μg/ml. The *rpsL* genotype of different *Bartonella* strains was selected with streptomycin ad 100 μg/ml, and ciprofloxacin resistance was selected with 1 μg/ml of that antibiotic.

### Plasmid construction

Plasmids were constructed using standard techniques of restriction-based molecular cloning. Point mutations and deletions were introduced into suitable template plasmids by PCR as described previously [57]. Plasmid construction was always confirmed by DNA sequencing. Molecular cloning was generally performed using *E*. *coli* Novablue, but vectors encoding a *ccdB* cassette were handled in *E*. *coli* DB3.1 that is genetically resistant to the action of the CcdB toxin. This study reports the construction of the pBZ485 broad-host range expression plasmid and other plasmid tools like the pAH182 suicide vector that will be useful tools for future work. The construction of these plasmids and additional experiments to validate their use is described in detail in S1 Text. An overview of the construction of all plasmids can be found in Table A in S1 Text.

### Strain construction

The construction of RP4 donor strain JKE201 is described in S1 Text. *B*. *schoenbuchensis* R1 had been isolated from the blood of wild roe deer [58]. In order to trace the transmission of pVbh_BscR1, we tagged it with a gentamicin resistance cassette using a Himar1 transposon delivered from suicide plasmid pML001 (inserted outside of the *dtr* locus of the Vbh machinery in a non-coding region) [59], creating *B*. *schoenbuchensis* R1 pVbh#2. *B*. *henselae* Houston-1 strain RSE247 is a commonly used *Bartonella* lab strain, and its derivative MQB759 is a mutant deficient in the production of BaGTA particles that can mediate interbacterial transfer of chromosomal markers [60]. A ciprofloxacin-resistant mutant of MQB759 was selected by streaking the bacteria on blood agar plates containing 0.4 μg/ml ciprofloxacin. Sequencing of the *gyrA* gene of the clone used in this study revealed a GAT to AAT transition in codon 95, encoding a D-to-N mutation in GyrA that is known to confer ciprofloxacin resistance in *Bartonella* [61]. Scarless deletion mutants in *vbhB4* and *traA* of pVbh_BscR1 were generated using suicide plasmids pPE3001 and p3007 through two-step homologous recombination as described previously [60, 62]. Plasmids were generally moved into *Bartonella* by conjugation from JKE201 or other RP4 donor strains. All bacterial strains used in this study are listed in Table 1.

**Table 1:** List and construction of all bacterial strains of this study.

Strain	Genotype	Source / Description / Construction
***Escherichia coli***		
Novablue	*endA1 hsdR17 (r*_*K12*_^*–*^ *m*_*K12*_^*+*^*) supE44 thi-1 recA1 gyrA96 relA1 lac F′[proA*^*+*^*B*^*+*^ *lacI*^*q*^*Z*Δ*M15*::*Tn10]*	our laboratory collection; standard cloning strain
DB3.1	F^-^ *gyrA462 endA1 glnV44* Δ*(sr1-recA) mcrB mrr hsdS20(r*_*B*_^*-*^, *m*_*B*_^*-*^*) ara14 galK2 lacY1 proA2 rpsL20 xyl5 Δleu mtl1*	our laboratory collection; CcdB-resistant cloning strain
W1872	F^+^ λ^–^	Coli Genetic Stock Center; harbors wildtype F-plasmid
*E*. *coli* K-12 MG1655	F^−^λ^−^*ilvG*^−^*rfb-50 rph-1*	our laboratory collection; K-12 wildtype strain
BW25113	F^-^ Δ*(araD-araB)567 ΔlacZ4787(*::*rrnB-3*) λ^-^ *rph-1* Δ*(rhaD-rhaB)568 hsdR514*	our laboratory collection; commonly used lab strain derivative of K-12 MG1655 (deficient in EcoKI restriction)
MFDpir	*RP4-2-Tc*::*[ΔMu1*::*aac(3)IV-ΔaphA-Δnic35-ΔMu2*::*zeo] ΔdapA*::*(erm-pir) ΔrecA*	Didier Mazel [63]; conjugation donor strain with chromosomal RP4 T4SS (derivative of K-12 MG1655)
JKE170	MFDpir Δ*mcrA* Δ*(mrr-hsdRMS-mcrBC)*	this study; derivative of MFDpir lacking all type IV restriction systems
JKE201	MFDpir Δ*mcrA* Δ*(mrr-hsdRMS-mcrBC) aac(3)IV*::*lacI*^*q*^	this study; derivative of MFDpir lacking EcoKI, the three type IV restriction systems, restored gentamicin sensitivity, harboring *lacI*^*q*^ allele
***Bartonella henselae***		
*B*. *henselae* Houston-1 RSE247	*rpsL*	our laboratory collection [60]; commonly used *B*. *henselae* laboratory strain
… MQ759	*rpsL* Δ*(BH13960-BH13970)*	our laboratory collection [64]; derivative of RSE247 deficient in production of BaGTA particles
… AHB0110	*rpsL* Δ*(BH13960-BH13970) gyrA(G283A)*	this study; ciprofloxacin-resistant derivative of MQ759 (carrying D95N mutation in GyrA)
… AHB0130	*rpsL* Δ*(BH13960-BH13970) gyrA(G283A) CRAfT_v8*.*1*	this study; pool of ca. 50 colonies from a transposition of CRAfT sensor transposon CRAfT_v8.1 into AHB0110
… MFE137	*rpsL* Δ*trwE*, Δ*virB2-11*	our laboratory collection [65]; derivative of RSE247 deficient in VirB T4SS and Trw T4SS
… AHB0127	*rpsL* Δ*trwE*, Δ*virB2-11* pVbh#2 pAH183_cre	this study; derivative of MFE137 harboring pVbh#2 and a plasmid encoding the Cre control for CRAfT
… AHB0128	*rpsL* Δ*trwE*, Δ*virB2-11* pVbh#2 pAH183_cre-traA	this study; derivative of MFE137 harboring pVbh#2 and a plasmid encoding the Cre-TraA relaxase fusion for CRAfT
… AHB0129	*rpsL* Δ*trwE*, Δ*virB2-11* pVbh#2 pAH183_cre-vbhT(H136A)	this study; derivative of MFE137 harboring pVbh#2 and a plasmid encoding the Cre-VbhT(H136A) fusion for CRAfT
… PEE0403	*rpsL* Δ*trwE*, Δ*virB2-11* pCD353	this study; derivative of MFE137 carrying pCD353
***Bartonella schoenbuchensis***		
*B*. *schoenbuchensis* R1 CHDE366	pVbh	our laboratory collection [58]; wildtype strain
… MLE0119	pVbh#2	this study; derivative of CHDE366 with gentamicin resistance cassette on pVbh (“pVbh#2”)
… PEE0352	*rpsL*; pVbh#2	this study; spontaneous streptomycin-resistant mutant of MLE0119
… PEE0370	*rpsL*; pVbh#2 Δ*vbhB4*	this study; derivative of PEE0352 with scarless deletion of *vbhB4*
… PEE0436	*rpsL*; pVbh#2 Δ*traA*	this study; derivative of PEE0352 with scarless deletion of an N-terminal part of the TraA relaxase

### DNA and protein sequences

We recently reported the genome sequence of *B*. *schoenbuchensis* R1 with genbank accession number CP019790.1 for pVbh_BscR1 [11], encoding the VbhT toxin with UniProt identifier E6Z0R3 (VBHT_BARSR). The UniProt identifiers of other previously described FicT toxins and related proteins are A1JNF3 (A1JNF3_YERE8; YeFicT), Q9I3X8 (Q9I3X8_PSEAE; PaFicT), and P20605 (FIC_ECOLI; EcFicT). The UniParc identifiers of single-domain FicT toxins of *Bartonella* are UPI00015FA8A2 (BtrFicT of *B*. *tribocorum* CIP 105476), UPI00026E5C06 (BelFicT of *B*. *elizabethae* ATCC 49927), and UPI00026E6E87 (BbiFicT of *B*. *birtlesii* LL-WM9). Plasmid pMETD1 of *M*. *extorquens* DM4 has genbank accession number NC_012987 and encodes the FicT toxin studied in Fig 6 and S3 Fig at locus_tag p1METDI0123 (UniProt identifier C7CN81 (C7CN81_METED)). Plasmid1 of *Chelativorans* sp. BNC1 has genbank accession number CP000389.1, and the PezT toxin studied in Fig 6 is encoded at locus_tag Meso_4336 (UniProt identifier Q11MR3 (Q11MR3_CHESB)). The rhizobial FIC domain proteins with a Bep-like β-hairpin shown in Fig 6A have UniProt identifiers B9K658 (B9K658_AGRVS; *Agrobacterium vitis*), B2IE13 (B2IE13_BEII9; *Beijerinckia indica*), A6X7M7 (A6X7M7_OCHA4; *Ochrobactrum anthropi*), and Q92N52 (Q92N52_RHIME; *Sinorhizobium meliloti*).

### Sequence analyses

Protein sequences were aligned using ClustalW or MAFFT implemented in Geneious v9.1.5 and manually curated. Interpro (https://www.ebi.ac.uk/interpro/) was used to annotate protein domains with the exception of BID domains that were identified using local BLAST searches with a sample of known BID domains as described previously [11]. The annotation of genes on pVbh (Fig 3 and S1 Fig) was based on BLAST searches and the identification of distant homologs using the Phyre2 protein homology modeling tool [66].

### AMPylation assays and protein expression

The AMPylation activity of VbhTA and BtrFicTA was assayed using cleared lysates of ectopically expressing *E*. *coli* as described previously [21]. In short, cleared lysates of *E*. *coli* cultures from expression of TA module constructs and target candidates (or empty vectors) were mixed with α-^32^P-ATP (Hartmann Analytic) to specifically label AMP transfer. Reactions were resolved by SDS-PAGE and protein AMPylation was visualized by autoradiography. The expression of BtrFicTA constructs required modifications in the design of protein expression constructs that are described in detail in S1 Text.

### pVbh conjugation experiments

Conjugation experiments assessing the transfer of pVbh_BScR1 were set up using *B*. *schoenbuchensis* R1 pVbh#2 or mutant derivatives as donors and *B*. *henselae* Houston-1 MFE137, a derivative of the RSE247 laboratory strain deficient in any type IV secretion, as recipients. The *B*. *henselae* strain harbored plasmid pCD353, encoding kanamycin resistance, to enable selection for recipients and transconjugants. Bartonellae were cultured for 4–7 days on CBA or HIA blood agar plates prior to experimentation. Per mating, the bacteria from one third of an agar plate of donors and one full agar plate of recipients were resuspended separately in each 1 ml of M199 (medium M199, Gibco, Invitrogen) supplemented with 10% FCS (fetal calf serum, Amimed) pre-warmed to 35°C and washed once in this medium. The OD600 of all samples was determined, and donors were added to recipients in a way that at least a ratio of 1: 5 was achieved. Bacterial suspensions were spun down, taken up in 120 μl of M199 + 10% FCS, and spotted onto a nitrocellulose filter on an HIA plate. Matings were incubated at 35°C / 5% CO_2_ for six hours. In parallel, serial dilutions of the donor samples were spotted on an HIA plate supplemented with gentamicin ad 10 μg/ml in order to quantify the number of donors per mating. After the mating, the bacteria were taken up in 1 ml of M199 + 10% FCS, washed once, and finally resuspended in 120 μl of the same medium. Serial dilutions were spotted on HIA plates containing kanamycin ad 30 μg/ml (to select for recipients) with or without gentamicin ad 10 μg/ml (to detect transfer of pVbh#2). Plates were incubated at 35°C / 5% CO_2_ for up to ten days. The frequency of plasmid transfer was calculated as the ratio of transconjugants per donor.

### CRAfT assay (*Escherichia coli*)

In this study we constructed and used a new variant of CRAfT that can be easily used in various bacterial organisms (see S1 Text) and that we applied successfully for *E*. *coli* (this section) and *Bartonella* (see below). Our system is based on the expression of Cre-fusions of candidate proteins in donor cells from a non-mobilizable derivative of pBZ485 and a CRAfT sensor module in recipient cells that can be delivered as a Himar1 transposon and detects transfer of Cre-fusions as a switch from spectinomycin to kanamycin resistance (see S1 Text).

For *E*. *coli*, recipients were derivatives of *E*. *coli* K-12 MG1655 or its BW25113 variant obtained from transposition of the CRAfT sensor transposon after delivery of suicide plasmid pAH182_CRAfT. Pools of 50 colonies from the transposition were used. CRAfT donors were *E*. *coli* JKE201 (carrying a chromosomal RP4 conjugation system; see S1 Text) harboring pAH188 (to assay conjugative plasmid transfer) and a vector encoding either the Cre recombinase alone or a translation fusion to the TraI relaxase of the RP4 machinery. For the CRAfT matings, donors were subcultured 1:100 for 3 hours and the expression of Cre fusion constructs was induced after 2 hours with IPTG ad ad 75 μg/ml. Bacterial samples were generated by washing 1.5 ml of recipient overnight cultures and 1 ml of donor subcultures separately in 1 ml of LB supplemented with 1 mM DAP and resuspending both samples in each 50 μl of LB supplemented with 1 mM DAP. Matings were set up by mixing donor and recipient samples and spotting the suspension on nitrocellulose filters (Sartorius) onto an LB agar plate supplemented with 1 mM of DAP. Matings were incubated at 37°C for 4 hours. Subsequently, bacteria were harvested, washed once in 1 ml of sterile PBS, and finally resuspended in 100 μl of sterile PBS. Serial dilutions of these samples were spotted onto LB agar plates containing different antibiotic combinations to select for all recipients that are / are not CRAfT recombinants, all transconjugants that are / are not CRAfT recombinants, all recombinants, and all donors. Conjugation of pAH188 was detected through chloramphenicol resistance, while CRAfT recombination was detected as a switch from spectinomycin to kanamycin resistance. Agar plates were incubated at 37°C for 24 hours, and the frequency of protein or plasmid transfer was calculated as the ratio of transconjugants or recombinants per donor (matings had more than 10x excess of recipients).

### CRAfT assay (*Bartonella*)

CRAfT recipients were created in the background of *B*. *henselae* Houston-1 MQB759, a derivative of the RSE247 lab strain that is impaired in the intercellular exchange of chromosomal markers via the *Bartonella* gene transfer agent (BaGTA) [64]. A ciprofloxacin-resistant mutant of this strain was selected in order to obtain an antibiotic resistance marker for CRAfT recipients (see above). Similar to our procedure for *E*. *coli*, a pool of 50 colonies obtained from transposition of the CRAfT sensor transposon from pAH182_CRAfT were used for the experiments (stock AHB0130; see Table 1). CRAfT donors were created by conjugating *cre* fusion plasmids into *B*. *schoenbuchensis* R1 pVbh#2 from *E*. *coli* JKE201. For VbhT, we used a catalytically inactive mutant (H136A [34]) in order to avoid possible effects of ectopic toxin expression on the translocation assay.

*Bartonella* donors and recipients were cultured on HIA plates for three to four days prior to experimentation. Per mating, the bacteria from one third of an agar plate of donors and three quarters of an agar plates of recipients were resuspended separately in each 1 ml of M199 + 10% FCS pre-warmed to 35°C and washed once in this medium. The OD600 of all samples was determined, and donors were added to recipients in a way that at least a ratio of 1: 5 was achieved. Matings were spun down, taken up in 120 μl of M199 + 10% FCS, and spotted onto a nitrocellulose filter on an HIA plate. While matings were incubated at 35°C / 5% CO_2_ for two hours, serial dilutions of the donor samples were spotted on a HIA plate supplemented with kanamycin ad 30 μg/ml in order to unambiguously quantify the number of donors per mating. After the mating, the bacteria were taken up in 1 ml of M199 + 10% FCS containing ciprofloxacin ad 1 μg/ml and agitated slowly for six hours at 35°C. After this period of outgrowth to enable efficient Cre/*loxP* recombination and phenotypic expression of resistance cassettes, the bacteria were washed in 1 ml of M199 + 10% FCS and finally taken up in 120 μl of the same medium. Serial dilutions were spotted on HIA plates containing ciprofloxacin ad 1 μg/ml and different antibiotic combinations to select for all recipients, all transconjugants, all recombinants, or all transconjugants that were also recombinants. Conjugation of pVbh#2 was detected as gentamicin resistance of recipients, while CRAfT recombination was detected as a switch from spectinomycin to kanamycin resistance. Plates were incubated at 35°C / 5% CO_2_ for ten days. The frequency of protein or plasmid transfer was calculated as the ratio of transconjugants or recombinants per donor.

## Supporting information

S1 Fig Detailed annotation of all genes on pVbh of *B. schoenbuchensis* R1. The annotation of all genes on pVbh is shown in detail. We colored the genes as described for Fig 3, i.e., in green for the Vbh conjugation system, light blue for plasmid replication and partitioning functions, red for TA modules, and light pink or dark pink for genes encoding proteins of unknown function with or without known protein domains, respectively.
(TIF)

S2 Fig Detection of protein transfer through the F conjugation system with the new CRAfT variant. *E*. *coli* BW25113 harboring pAH204, an F-plasmid derivative encoding chloramphenicol resistance, or control plasmid pBAD33 were transformed with vectors to express Cre or Cre fused to the TraI relaxase of the F conjugation system. The CRAfT assay was performed similar as for RP4, but instead of 1 ml of exponentially growing donors, 150 μl of overnight culture were used. Liquid culture matings were set up by mixing donors and recipients in a final volume of 200 μl of LB supplemented with 1mM DAP and incubated at 37°C for 2.5 hours. Our results showed that even the weak basal expression of the Cre-TraI fusion substantially impaired conjugative transfer of pAH204 (blue bars), as expected from the literature [67]. Nevertheless, we could detect translocation of the Cre-TraI fusion protein (green bars) at every successful conjugative plasmid transfer (blue / green mixed bar). Data points and error bars represent mean and standard deviation of three independent experiments.
(TIF)

S3 Fig Additional data related to Fig 6. (A) Multiple sequence alignment of different FicT proteins centered on the region containing the β-hairpin of unknown function that was previously considered to be Bep-specific (green; [11]). Note that this hairpin is also found in a small group of rhizobial FicT toxins closely related to the Beps (Fig 6A). The sequence alignment also highlights the flap β-hairpin that is involved in target interaction (red) and the FIC signature motif at the active site (blue). (B) A deeper phylogeny of FicT toxins provides additional support for the independent recruitment of different representatives as T4SS substrate (compare Fig 6A). The Maximum Likelihood phylogeny was generated using PhyML from multiple sequence alignment generated with MAFFT (both implemented in Geneious v10.1.3) and with parameters optimized with the help of ProtTest 3 [68]. Bootstrap support (of 100) is shown if >80. The strong support of phylogenetic groups containing separate instances of demonstrated or *bona fide* T4SS substrates is highlighted in red. The UniProt identifiers of proteins not listed in the corresponding section of *Materials and Methods* are Q9PCU8 (Q9PCU8_XYLFA; FicT of *Xylella fastidiosa*), H1PYN1 (H1PYN1_9FUSO; FicT of *Fusobacterium ulcerans*), C0D2F7 (C0D2F7_9FIRM; FicT of *Clostridium asparagiforme*), and E2CSK7 (E2CSK7_9RHOB; FicT of *Roseibium* sp. TrichSKD4). FicT of *Pelagibacterium luteolum* has NCBI accession number WP_090598185. (C) Protein sequence alignment of *B*. *schoenbuchensis* R1 VbhT (UniProt identifier E6Z0R3 (VBHT_BARSR)) and *M*. *extorquens* DM4 FicT (UniProt identifier C7CN81 (C7CN81_METED)). The two proteins share 27.4% sequence identity in their FIC domain (interpro IPR003812) and 19.6% sequence identity in the region that aligns to the BID domain of VbhT. (D) Protein sequence alignment of TraA relaxases of *B*. *schoenbuchensis* R1 pVbh (UniProt identifier E6Z0R5 (E6Z0R5_BARSR)) and *M*. *extorquens* DM4 p1METDI (UniProt identifier C7CN79 (C7CN79_METED)). The two proteins share 36.6% sequence identity in their MobA/MobL domain (interpro IPR005053) and 42.6% sequence identity in their P-loop ATPase domain (interpro IPR027417) that are core of actual relaxase function, but only 19.6% in the region that aligns to the BID domain of pVbh TraA.
(TIF)

S1 Table List of oligonucleotide primers used in this study. (PDF)

S1 Text List / construction of plasmids used in this study and *E. coli* strain JKE201. (PDF)
