# Supplementary figures and images for: A bacterial toxin-antitoxin module is the origin of inter-bacterial and inter-kingdom effectors of Bartonella
Source: PLoS Genet. 2017 Oct 26;13(10):e1007077. doi: 10.1371/journal.pgen.1007077 (PMC5675462; doi:10.1371/journal.pgen.1007077)

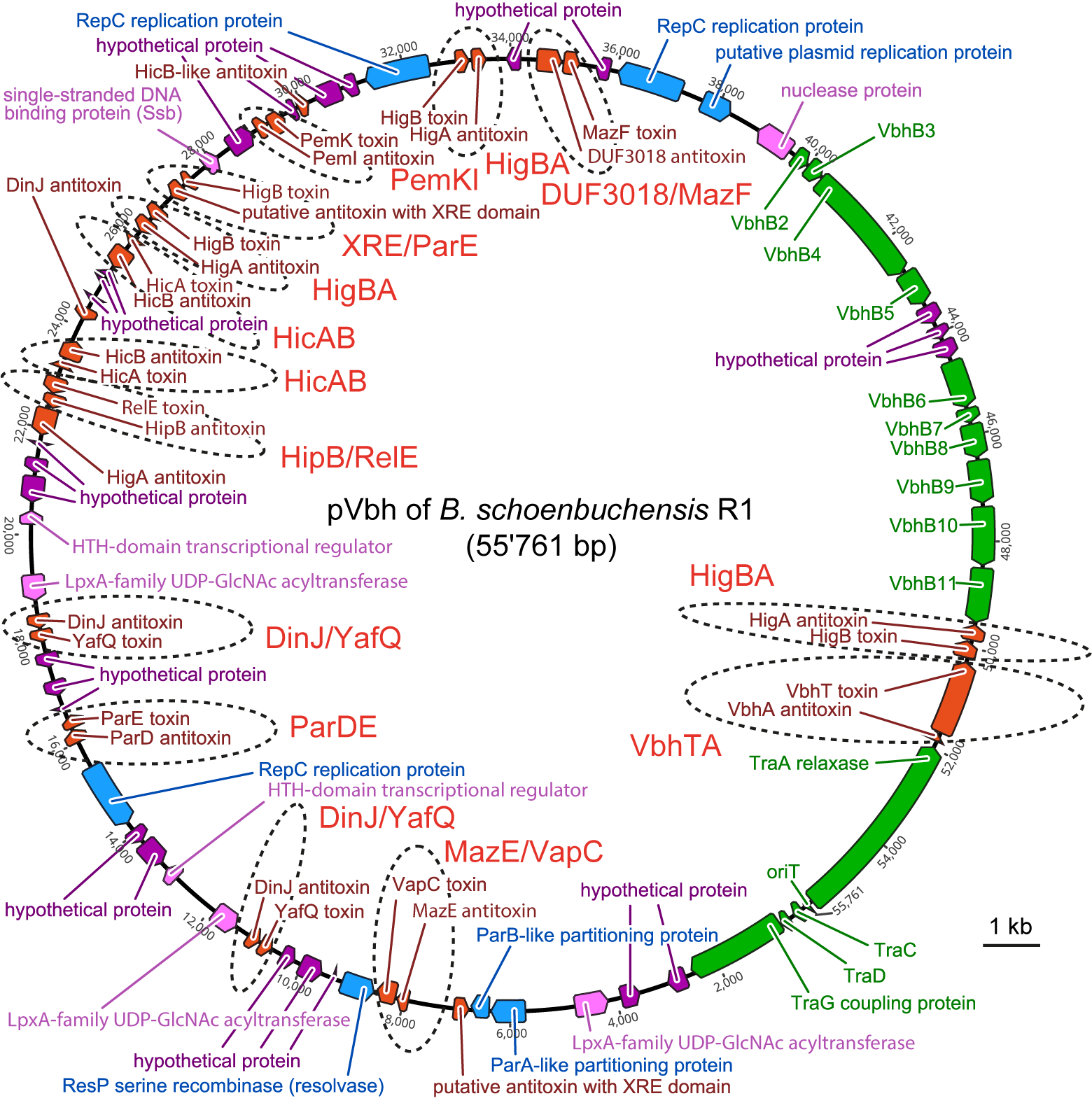

Supplement: S1 Fig — The annotation of all genes on pVbh is shown in detail. We colored the genes as described for Fig 3, i.e., in green for the Vbh conjugation system, light blue for plasmid replication and partitioning functions, red for TA modules, and light pink or dark pink for genes encoding proteins of unknown function with or without known protein domains, respectively. (TIF) [file pgen.1007077.s001.tif]

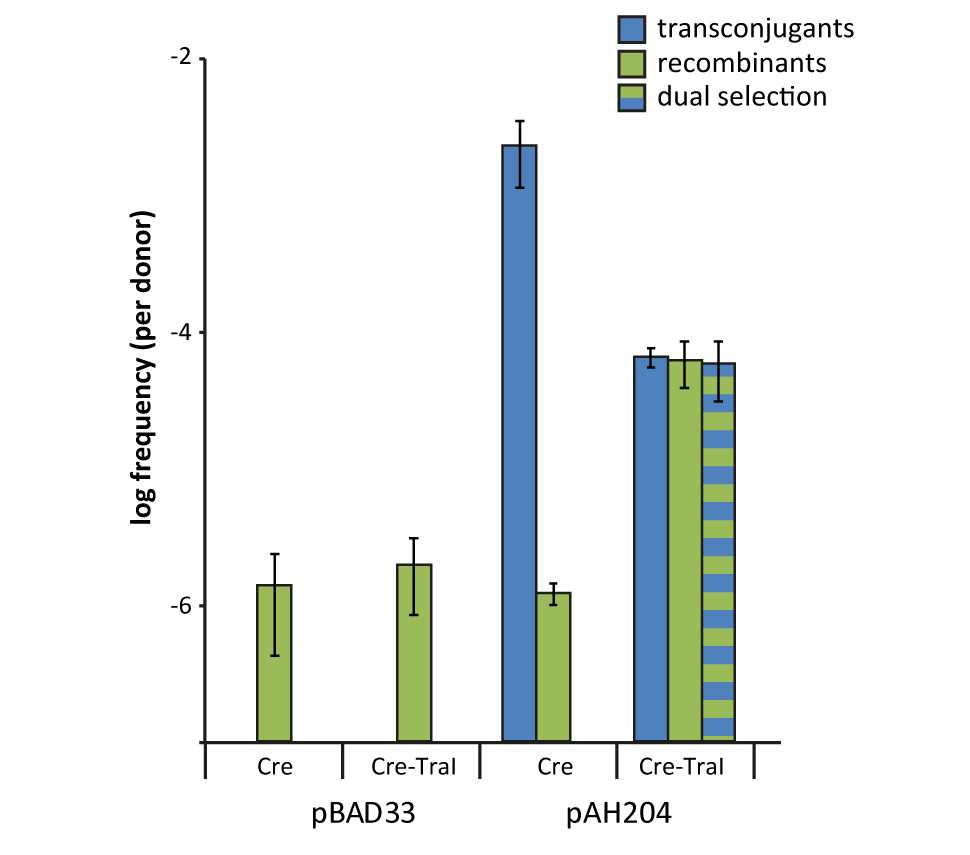

Supplement: S2 Fig — E. coli BW25113 harboring pAH204, an F-plasmid derivative encoding chloramphenicol resistance, or control plasmid pBAD33 were transformed with vectors to express Cre or Cre fused to the TraI relaxase of the F conjugation system. The CRAfT assay was performed similar as for RP4, but instead of 1 ml of exponentially growing donors, 150 μl of overnight culture were used. Liquid culture matings were set up by mixing donors and recipients in a final volume of 200 μl of LB supplemented with 1mM DAP and incubated at 37°C for 2.5 hours. Our results showed that even the weak basal expression of the Cre-TraI fusion substantially impaired conjugative transfer of pAH204 (blue bars), as expected from the literature [67]. Nevertheless, we could detect translocation of the Cre-TraI fusion protein (green bars) at every successful conjugative plasmid transfer (blue / green mixed bar). Data points and error bars represent mean and standard deviation of three independent experiments. (TIF) [file pgen.1007077.s002.tif]

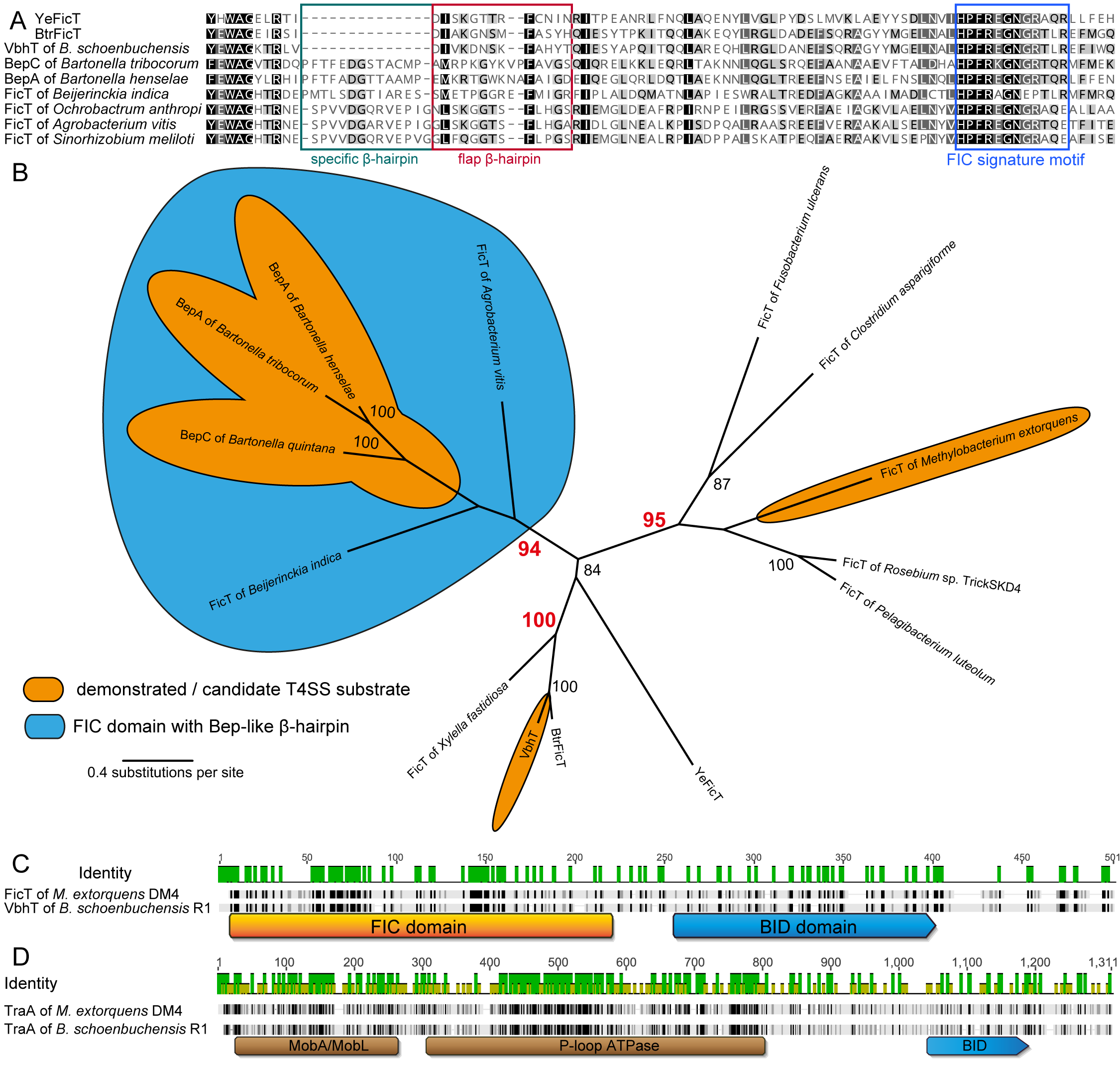

Supplement: S3 Fig — (A) Multiple sequence alignment of different FicT proteins centered on the region containing the β-hairpin of unknown function that was previously considered to be Bep-specific (green; [11]). Note that this hairpin is also found in a small group of rhizobial FicT toxins closely related to the Beps (Fig 6A). The sequence alignment also highlights the flap β-hairpin that is involved in target interaction (red) and the FIC signature motif at the active site (blue). (B) A deeper phylogeny of FicT toxins provides additional support for the independent recruitment of different representatives as T4SS substrate (compare Fig 6A). The Maximum Likelihood phylogeny was generated using PhyML from multiple sequence alignment generated with MAFFT (both implemented in Geneious v10.1.3) and with parameters optimized with the help of ProtTest 3 [68]. Bootstrap support (of 100) is shown if >80. The strong support of phylogenetic groups containing separate instances of demonstrated or bona fide T4SS substrates is highlighted in red. The UniProt identifiers of proteins not listed in the corresponding section of Materials and Methods are Q9PCU8 (Q9PCU8_XYLFA; FicT of Xylella fastidiosa), H1PYN1 (H1PYN1_9FUSO; FicT of Fusobacterium ulcerans), C0D2F7 (C0D2F7_9FIRM; FicT of Clostridium asparagiforme), and E2CSK7 (E2CSK7_9RHOB; FicT of Roseibium sp. TrichSKD4). FicT of Pelagibacterium luteolum has NCBI accession number WP_090598185. (C) Protein sequence alignment of B. schoenbuchensis R1 VbhT (UniProt identifier E6Z0R3 (VBHT_BARSR)) and M. extorquens DM4 FicT (UniProt identifier C7CN81 (C7CN81_METED)). The two proteins share 27.4% sequence identity in their FIC domain (interpro IPR003812) and 19.6% sequence identity in the region that aligns to the BID domain of VbhT. (D) Protein sequence alignment of TraA relaxases of B. schoenbuchensis R1 pVbh (UniProt identifier E6Z0R5 (E6Z0R5_BARSR)) and M. extorquens DM4 p1METDI (UniProt identifier C7CN79 (C7CN79_METED)). The two proteins share 36.6% s [file pgen.1007077.s003.tif]
