# Supplementary material for: A bacterial toxin-antitoxin module is the origin of inter-bacterial and inter-kingdom effectors of Bartonella
Source: PLoS Genet. 2017 Oct 26;13(10):e1007077. doi: 10.1371/journal.pgen.1007077 (PMC5675462; doi:10.1371/journal.pgen.1007077)
Supplement: S1 Table — (PDF) [file pgen.1007077.s004.pdf]

Table S1. List of all oligonucleotide primers used in this study

Primer name	*Sequence (5'-3')*
prAH402	GAGCGGAGATCTATGGCCTACCCATACGATGTTCCAGATTACGCGGCCGCGATGTTGAGTGAAGACGAAATTA
prAH403	CTCCCGCCTCGAGTTATATTTCTCCACGTTTTATCC
prAH404	GAGCGGCTGCAGGTGGAGAAATATAAAGGGGG
prAH405	CTCCCGCGCGGCCGCTTATAAGAGATTGAGGTTTTGAC
prAH443	ATGCCTTGGCCCCCTTTCGAGAGGGAAATG
prAH444	CGAAAGGGGGCCAAGGCATTCAACTCTCCC
prAH498	GAGCGGGGTACCATAGGAGGAACAATATGTTGAGTGAAGACGAAATTA
prAH499	CTCCCGCAAGCTTTTATATTTCTCCACGTTTTATCC
prAH523	GAGCGGGGTACCATAGGAGGAACAATTTTGTGGAGAAATATAAAGGGGG
prAH524	CTCCCGCCTCGAGTTATAAGAGATTGAGGTTTTGAC
prAH547	AGATATACATATGGCCTACCCATACGATGTAGAT
prAH548	GGTAGGCCATATGTATATCTCCTTCTTATACTTA
prAH939	GAGCGGTCTAGAGCTCATAGGAGGAACAATATGCAGTTTAAGGTTTACACCTATAAA
prAH940	CTCCCGCAAGCTTGCGGCCGCTCGAGGTACCATCGATTATATTCCCCAGAACATCAGG
prAH1113	ATGTATTTAGCCATGTCAGCCGTTAAGTG
prAH1114	CATGGCTAAATACATTCAAATATGTATCCGCT
prAH1115	GAGCGGCCTGCAGGTAGCGATAGCGGAGTGTATACT
prAH1116	CTCCCGCGGTACCGTTCAGTAATTTCCTGCATTTG
prAH1117	GAGCGGCAATTGTAGGAGCACAAAATGTCCAATTTACTGACCG
prAH1118	CTCCCGCGAATTCGCTGCCACCGCCACCGCTATCGCCATCTTCCAGC
prAH1123	AACAGCGAGGCAGCATGAAGAACGACAGGACTTTG
prAH1124	CTGCCTCGCTGTTGCACAAAGTAGGGTCGGGATT
prAH1181	GAGCGGGAATTCATTGCCAAGCACGTCC
prAH1182	CTCCCGCCTCGAGCAATTGTCATCTACTCCTACCTCGGG
prAH1238	GACGCCATGCATCAGGAGGGACAGCTGATAG
prAH1239	CTCGGGCCTGCAGCGGGAATTTGAAGACAATAAC
prAH1240	GACGCCCTGCAGGAAAGTTCTTTTACCCTCAGC
prAH1241	CTCGGCGATGCATGCGGATACATATTTGAATGTATTTAGA
prAH1242	GACGCCGTCGACTCACTGCCCGCTTTC
prAH1243	GACGCCCTGCAGTATGAGTCAGCAACACCTTCT
prAH1244	GAACGGCCTCGAGCGCACAAGTCCTAAGTTACG
prAH1245	CATCGATTAATCGCCATCTTCCAGCA
prAH1246	TGGCGATTAATCGATGGTACCTCGAGC
prAH1247	GACAGGGGAATTCGCAATAGCGCATTTATCAGC
prAH1248	CTCCCGCCTCGAGTTACCTTGTAATTCCCTTTGAAGG
prAH1249	GAGCGGCGAATTCAGGAAATATGAGGGTAGCAACG
prAH1267	GATAGGTGAAGTAGGCCCACCCGCGAGCGGGTGTTCCTTCTTCACTGTCCCTTATTCGGTTTTCCCAGTCACGAC
prAH1268	CGCGGGTGGGCCTACTTCACCTATCCTGCCCGGCTGACGCCGTTGGATACACCAAGGAAAGTCTACAGCGTAATAGCGAAGAGGC
prAH1300	CCGCTGGACTCGAGTTATTGAACAAGATGGATTGCA
prAH1301	CTCCCGGAATTCCATGCGAAACGATCCTC
prAH1302	GAGCGGGAATTCATTACCTCATATAGCATACATTATACGAAGTTATCGTAGAAGAACAGCAAGGC
prAH1303	CTCCCGCTCGAGATAACTTCGTATAATGTATGCTATACGAAGTTATCTATTGCGATAACAAGAAAAAGC
prAH1321	GGTGTGGACCTGCAGGCTAAAGGAAGCGGAACAC
prAH1322	CTCCCAGCGGCCGCGGAACTTCAAGATCCCCTTATTAG

prAH1393	ACGAATTCCCGTCGTGACTGGGAAAACC
prAH1394	GTCACGACGGGAATTCGTAATCATGGTC
prAH1435	GAACTCGGAATTCAGTATTGCGCAGGTCAGAT
prAH1436	CTCCGGTCTCGAGCGTTTGTCTTCCTGTATCAGTC
prAH1441	ACAAGTAATGTGCGCACACTACATCCCTGATACGAACAAAGTTAACTTATCAGGAGGGACAGCTGATAG
prAH1442	CAGGTTGACTTTTCAGAATAGGGGCAGTTTGGTGATCGTCACCAGTATATCGGGAATTTGAAGACAATAAC
prJK071	GGAGCGGTATTTTCTATAAAACTTACCGCTTATTTGAGATATTCATCGAAATTCCGGGGATCCGTCGACC
prJK072	TAAGGCGCAGCCGCATCCGGCCTGATATTTCACTCAAAATAGTCCATATCTGTAGGCTGGAGCTGCTTCG
prJK073	TCAATAAAAGTAGTATTGTCGTGAAAAATTGATTAAAGATTAATATTATGATTCCGGGGATCCGTCGACC
prJK074	CGTCTTAAATGTGACTTTAATAAATAATTTTATTTCTGTAATCGGTTTATTGTAGGCTGGAGCTGCTTCG
prJK106	ACTATTTGCAACAGTGCCGTTGATCGTGCTATGATCGACTGATGTCATCACGACATCATAACGGTTCTGG
prJK110	TCTGCTGTAGTGAGTGGGTTGCGCTCCGGCAGCGGTCCTGATCATGAGCTGGAATCGAAATCTCGTGATG
prJK111	ACTATTTGCAACAGTGCCGTTGATCGTGCTATGATCGACTGATGTCATCACACAGCCATACCACAGCTTC
prJK112	TCTGCTGTAGTGAGTGGGTTGCGCTCCGGCAGCGGTCCTGATCATGAGCTCTTACATTAATTGCGTTGCG
prMQ1681	TAACAATATGTTGTGTCGAATTGTGAGCGGATAACAATTTCTCACAGGAAACAGCTATGACC
prMQ1682	CCGCTCACAATTCGACACAACATATTGTTATCCGCTCACAAGTAAAGCCTGGGGTGCCTAG
prPE506	CGGGATCCAACTTCGCCGGCAGTTTGTG
prPE507	ATGAGGAAAATTTTACTAACAATAG
prPE508	GTTAGTAAAATTTTCCTCATTTTAGCTCCTCATAATTACGG
prPE509	CGGGATCCTGAATGCTATGACGGGAACC
prPE511	AAGGTAAAATTCTTTTAAACGAT
prPE609	CGGGATCCTGTTGATTGTTATCTCAACAAG
prPE610	ATCGTTTAAAAGAATTTTACCTTTAACCTGATATTTTAGCTAAAGCGC
prPE611	GCGGATCCGTTTTATCCGTTCCATCAAATCC
