# Supplementary material for: A bacterial toxin-antitoxin module is the origin of inter-bacterial and inter-kingdom effectors of Bartonella
Source: PLoS Genet. 2017 Oct 26;13(10):e1007077. doi: 10.1371/journal.pgen.1007077 (PMC5675462; doi:10.1371/journal.pgen.1007077)
Supplement: S1 Text — (PDF) [file pgen.1007077.s005.pdf]

Expression of BtrFicTA for AMPylation assays

We previously expressed VbhTA constructs for lysate AMPylation assays from the pRSFDuet-1 backbone, a plasmid with two multiple cloning sites that each enable the expression of constructs from a P*T7* promoter. Our constructs encoded either the VbhT toxin alone (with an N-terminal hexahistidine (His6-)tag) to be expressed from the first multiple cloning site or had additionally the VbhA antitoxin (with an N-terminal hemagglutinin (HA-)tag) cloned into the second multiple cloning site [1]. However, we continuously failed to express soluble BtrFicT using the same procedure. Accidentally, we constructed a pRSFDuet-1 derivative encoding *His6-btrficT* in the first multiple cloning site and *HA-btrficA* in the second multiple cloning site in a way that an additional ATG start codon had been inadvertently placed between the ribosome binding site (RBS) and the ATG start codon of the HA-tag of *HA-btrficA*, but not in frame (plasmid pAH134b; see illustration below).

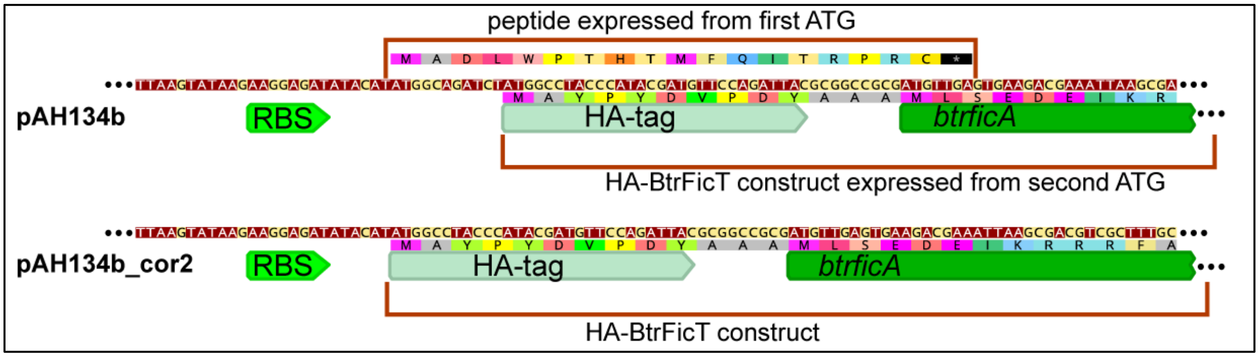

Two constructs to express BtrFicT and BtrFicA

The original BtrFicTA expression plasmid pAH134b (top) has an ATG directly behind the ribosome-binding site (RBS) ahead of the ATG start codon of the *HA-btrficA* construct. In consequence, a short peptide is primarily expressed and only a low amount of HA-BtrFicA is produced. This construct enables the expression of substantial amounts of soluble His6-BtrFicT from the other multiple cloning site. In a second construct (pAH134b_cor2, bottom) the first ATG has been removed, enabling parallel expression of His6-BtrFicT and HA-BtrFicA.

This plasmid therefore expresses only a marginal amount of HA-BtrFicA and caused considerable growth inhibition due to BtrFicT, but – curiously – enabled the expression of substantial amounts of soluble His6-BtrFicT. For this reason, we used plasmid pAH134b for the expression of functional BtrFicT. We speculate that low, sub-stoichiometric amounts of the BtrFicA antitoxin may transiently bind to hydrophobic patches on the surface of BtrFicT and thereby support protein folding. For the co-expression of BtrFicT and BtrFicA we used pAH134b_cor2 which is a derivative of pAH134b with the start codon of *HA-btrficA* placed closely downstream of the RBS of the second multiple cloning site. The construction of different *btrficTA* expression plasmids is described in short in Table A (see below).

Construction of conjugation donor strain JKE201

For a long time, *E. coli* strains SM10 and S17-1 had been widely used as donor strains for the mobilization of plasmids with RP4 origin of transfer (*oriT*) through their chromosomally encoded RP4 type IV secretion system (T4SS). However, a recent study indicated that these strains are not well suited for this purpose, because they harbor several active copies of the Mu prophage and a chromosomal RP4 *oriT* that enables the transfer of various chromosomal loci into recipient cells [2]. These authors therefore created *E. coli* MFDpir, an RP4 T4SS donor strain based on wildtype K-12 MG1655 that was devoid of these drawbacks. In this study, we created *E. coli* JKE201 as an improved variant of *E. coli* MFDpir.

Initially, we decided to improve the compatibility of MFDpir with the introduction of foreign methylation patterns (to overcome restriction barriers in recipient bacteria) by eliminating the McrA, McrBC, and Mrr type IV restriction systems of *E. coli* K-12 together with the EcoKI type I restriction system encoded by *hsdRMS* [3, 4]. The *mcrA* and *mcrCB-symER-hsdRMS-mrr* loci were deleted using lambda red recombineering with kanamycin resistance cassettes amplified from pKD13 and subsequent restoration of kanamycin sensitivity through recombination of flanking FRT sites [5]. The recombineering cassette for *mcrA* was amplified with primers prJK073 / prJK074 and the recombineering cassette for *mcrCB-symER-hsdRMS-mrr* was amplified with primers prJK071 / prJK072. Deletion of the two loci created strain JKE170. When using this strain for the delivery of different plasmids into *Bartonella*, we realized that it displayed resistance to gentamicin, likely caused by apramycin resistance cassette *aac(3)IV* that had been used to eliminate one copy of phage Mu during the construction of MFDpir [2, 6]. We therefore deleted the *aac(3)IV* cassette from JKE170 and replaced it with a *lacI*^{q} allele that enables a more efficient repression of P*lac* promoters. For this purpose, we applied a two-step recombineering procedure with a double-selectable cassette (kanamycin resistance / sucrose sensitivity) based on pJM05 as described previously [1]. The double-selectable cassette with flanking homologies for the deletion of *aac(3)IV* was amplified with prJK106 / prJK110, inserted into JKE170 through lambda red recombineering, and subsequently replaced with *lacI*^{q} that had been amplified from pBZ485 using prJK111 and prJK112. Resulting strain JKE201 is a versatile RP4 donor for the delivery of plasmids into various organisms and routinely used for this purpose by our laboratory.

Construction and validation of versatile broad-host range plasmid pBZ485

Background

In this study we report the construction of pBZ485, a versatile broad-host range expression plasmid that was initially constructed to overcome the need for a new generation of gene expression plasmids for *Bartonella*, but that should be useful in many different organisms. So far, gene expression in *Bartonella* had often been performed with derivatives of pCD341, a P*tac-lac* plasmid based on the broad-host range RSF1010 backbone that had been constructed more than 20 years ago [7]. Though this vector has been used successfully in many different studies, it suffered from three major drawbacks that were addressed during the construction of pBZ485: First, the P*tac-lac* of pCD341 was not very tightly controlled and had significant basal expression in the absence of the inducer IPTG [7]. Additionally, we had repeatedly observed that the presence of short repeats around the P*tac-lac* promoter of pCD341 occasionally resulted in deletion of part of the promoter sequence through recombination events. Furthermore, it had been shown or confidently inferred repeatedly that the DNA processing and transfer (Dtr) functions of the RSF1010 backbone interfere with the functioning of a wide range of type IV secretion machineries: Previous work showed that RSF1010 prevents substrate binding to the VirD4 coupling protein of the *Agrobacterium tumefaciens* VirB T4SS, suppressing virulence of this organism that depends on transfer of Ti-DNA and effectors into plant host cells [8]. A similar effect has been shown on the host-targeting Dot/Icm T4SS of *Legionella pneumophila* (blocking effector secretion and impairing host cell infection) [9] as well as the conjugative AvhB T4SS of *A. tumefaciens* [10]. Given that the AvhB T4SS is among the closest relatives of the Vbh and VirB T4SS machineries of *Bartonella* [11, 12], we were concerned that pCD341 derivatives may also affect the functioning of type IV secretion in *Bartonella* (although previous work did not report obvious effects of pCD341 at least on host cell phenotypes depending on the VirB T4SS of *B. henselae* [13]). We therefore created pBZ485 as a derivative of pCD341 featuring 1) a more tightly controlled IPTG-inducible promoter that lacks repeat sequences and 2) deletion of the original Dtr functions of RSF1010 that were replaced with a synthetic RP4 *oriT*. A short description of the construction of pBZ485 is also found in Table A (see below).

Construction and validation of pBZ485

For the optimization of promoter elements of pCD341 we used pCD353, a derivative of pCD341 encoding the green fluorescent protein (GFP) variant *gfpmut2* under control of P*tac-lac* [7]. When we tested different variants of this promoter that lacked internal repeats, we identified pCD353_v5 with P*lac*(MQ5) as a plasmid with tightly controlled expression of *gfpmut2* both in *Bartonella* and in *E. coli* (see

panel A of the illustration below). We therefore used this plasmid as the basis for a new general cloning vector and replaced the *gfpmut2* gene with a *ccdB* toxin cassette that enables convenient cloning via positive selection for clones lacking this toxin [14]. Additionally, we introduced a second MCS downstream of the *ccdB* cassette, so that it is flanked by two multiple cloning sites and can easily be deleted by directional cloning (see panel B of the illustration below).

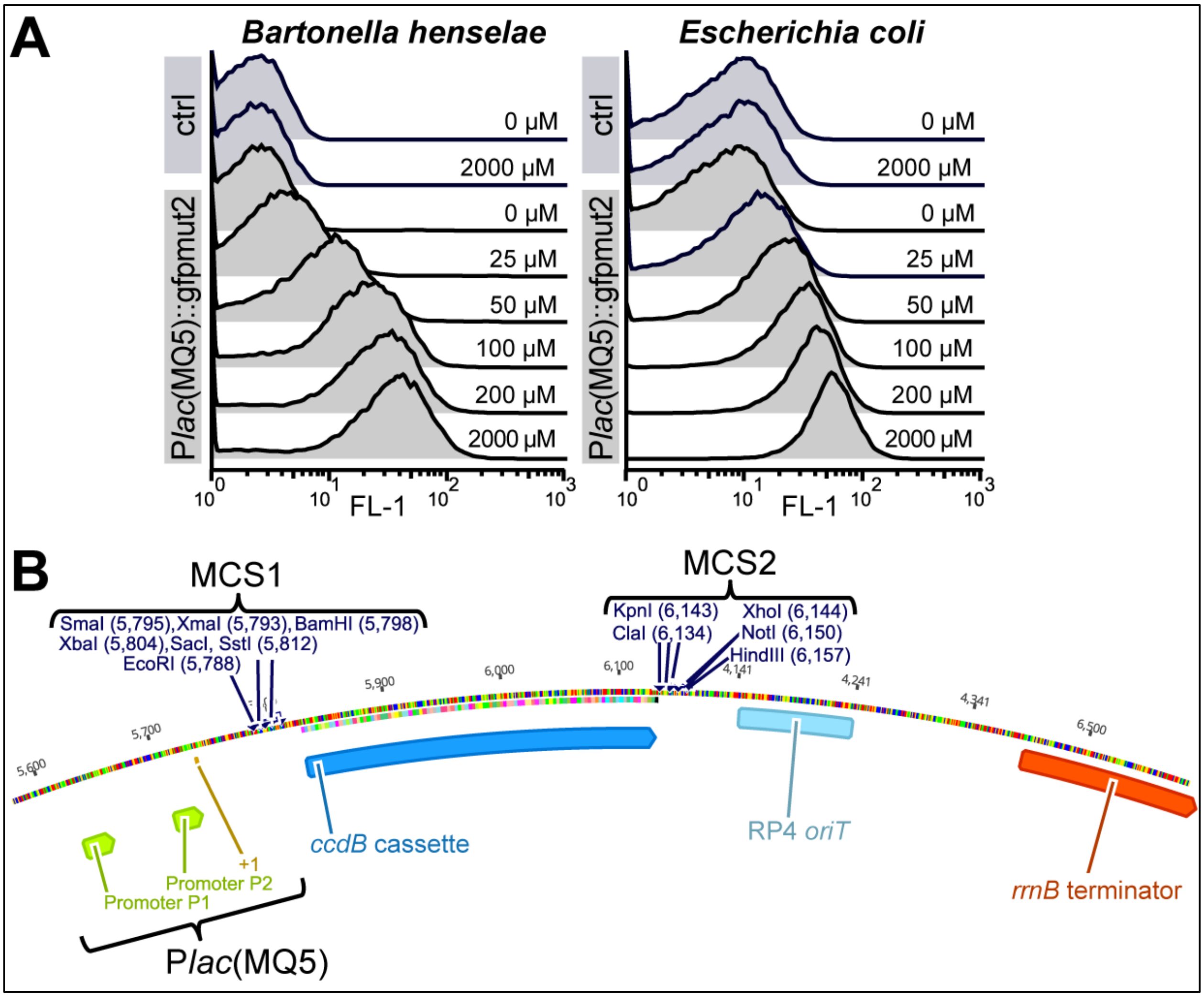

Schematic map of the pBZ485 MCS region and evaluation of P*lac*(MQ5) induction on single-cell level
(A) pCD353_v5 was probed for induction of GFP fluorescence with IPTG (see methodology below). Both in *Bartonella henselae* and *E. coli*, the vector exhibited no relevant induction compared to the promoterless control in the absence of IPTG. Addition of the inducer caused a dose-dependent response of GFP expression in the whole population. The experimental procedure is described in the dedicated section below. **(B)** Schematic map of the key region of pBZ485 showing the novel variant of Plac called Plac(MQ5), a *ccdB* cassette flanked by two multiple cloning sites (MCS1 and MCS2) to enable convenient, directional cloning with negative selection, the RP4 *oriT*, and an *rrnB* terminator.

Subsequently, we deleted potentially problematic Dtr components from the RSF1010 backbone. Generally, RSF1010 and related plasmids encode a conjugative relaxase as well as a primase that is translocated into conjugative recipients large relaxase-primase fusion protein [15]. In addition, the

primase is expressed as a short form that is essential for vegetative plasmid replication of RSF1010. Secretion of the primase improves RSF1010 conjugation into heterologous hosts likely by improving DNA processing in the recipient cell, while the short form cannot be secreted [16]. We therefore deleted a region on the RSF1010 backbone that spans from the *oriT* over the relaxase gene in a way that the primase gene will be transcribed from the promoter and ribosome-binding site of the relaxase-primase fusion (creating pBZ485a_Δdtr). In order to restore the ability of the plasmid to be mobilized through the RP4 conjugation system of donor strains like JKE201 (by the endogenous relaxase of RP4), we introduced a minimal *oriT* of the RP4 conjugation system that had been designed by the iGEM Registry (BioBrick part BBa_K125320). This *oriT* restored conjugation via RP4 at the same frequencies as original pCD353 and created plasmid pBZ485. A schematic map of plasmid pBZ485 is shown in the illustration below, and the different steps of its construction are summarized in Table A.

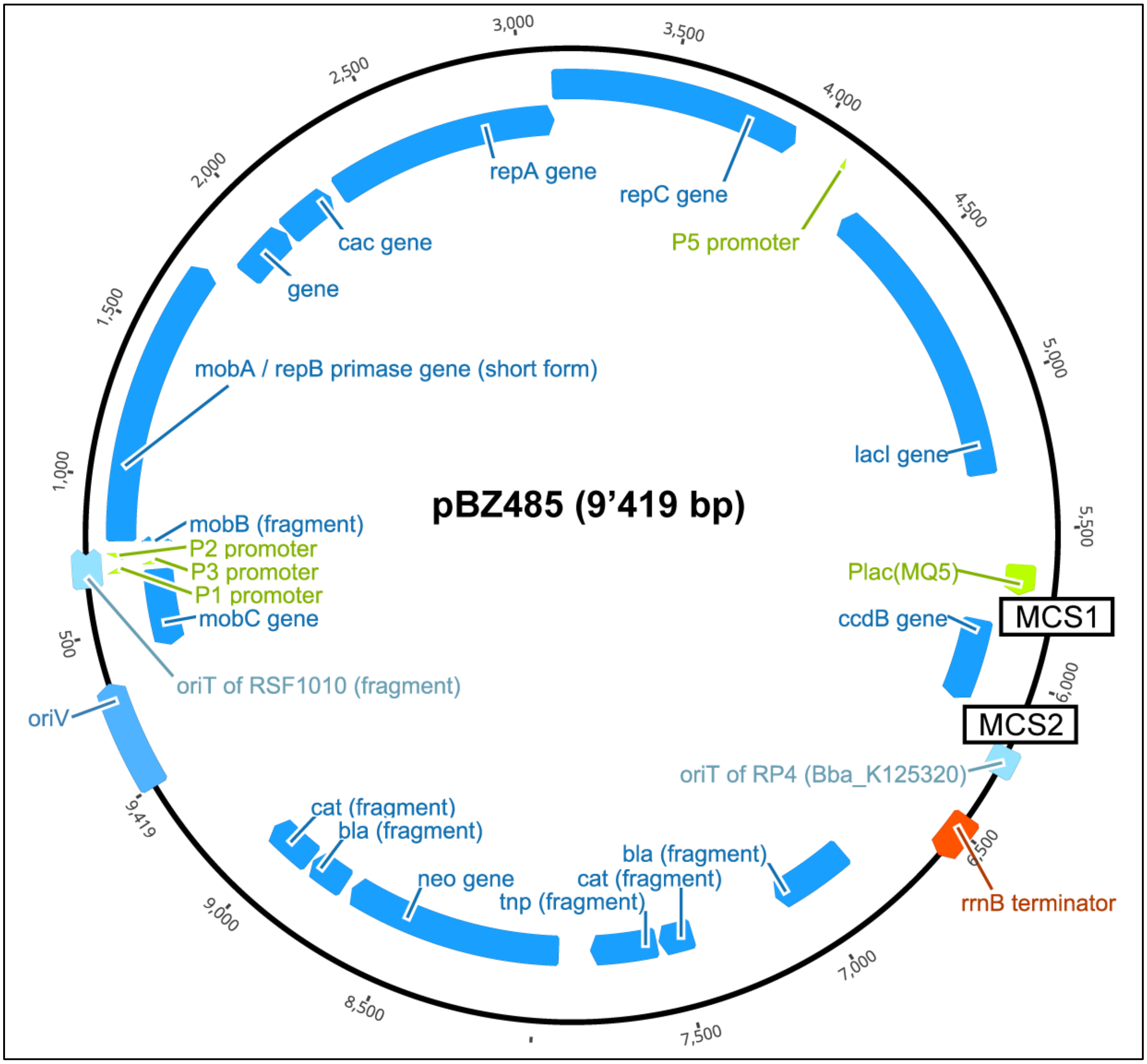

Schematic map of pBZ485

Analysis of P*lac*(MQ5) promoter activity by flow cytometry

Flow cytometry analysis of *B. henselae* strains carrying pCD353_v5 was performed as described previously for other transcriptional fusions of GFP in *Bartonella* [13, 17]. In short, bacteria were grown on blood agar plates supplemented with 30 µg ml^{-1} kanamycin at 35°C and 5% CO_2 for two days. Subsequently, bacterial samples were resuspended in M199 (Gibco) supplemented with 10% fetal calf serum (Animed) at a final OD_{600} of 0.008 and incubated in 48-well plates in a humidified atmosphere at 35°C and 5% CO_2 for 6 h. Expression of the *gfpmut2* reporter was measured as GFP fluorescence of c.a. 25’000 cells using a FACSCalibur flow cytometer (BD Biosciences) with an excitation at 488 nm. Data analysis was performed using the FlowJo software (Flowjo LLC). Similarly, the expression of GFP from

pCD353_v5 was probed in *E. coli* BW25113 by recording GFP fluorescence of ca. 25'000 cells per sample 2 h after the addition of IPTG inducer to exponentially growing cultures in LB medium at 37°C / 200 rpm.

Construction of a versatile variant of CRAfT

Background

Cre recombinase assay for translocation (CRAfT) is an assay for the detection of intercellular protein transfer based on the fusion of candidate proteins to the Cre recombinase in donor cells and the detection Cre-mediated site-specific recombination of *loxP* sites in recipient cells [18]. Different variants of CRAfT have been used previously to detect protein transfer through T4SS machineries into host cells [11, 18, 19] or into other bacteria [20, 21]. In this study we created a new variant of CRAfT with the aim to provide a tool that could be used to study interbacterial protein transfer in many different organisms without major adaptations of the experimental procedure. For this purpose, we designed the *loxP*-based recipient sensor module on a Himar1 transposon that can be delivered from a suicide plasmid using an RP4 T4SS donor *E. coli* strain into virtually any other microorganism. This CRAfT sensor module detects the translocation of Cre fusions as a switch from spectinomycin to kanamycin resistance, because Cre / *loxP* recombination removes a floxed spectinomycin resistance cassette that we had placed within a kanamycin resistance gene. Cre fusions are expressed in donors from a non-mobilizable variant of broad-host range plasmid pBZ485, enabling the plasmid to be used in many different organisms.

Construction of pAH182_CRAfT

We constructed suicide plasmid pAH182 for the delivery of our CRAfT sensor transposon on the backbone of well-known suicide plasmid pGP704 [22]. This plasmid carries an R6K *ori* that can only replicate in bacteria expressing the *pir* [23], gene, making it a suicide plasmid that can be used in wildtype *E. coli* as well as in other organisms. An RP4 *oriT* enables efficient delivery from JKE201 or another RP4 T4SS donor strain. We first eliminated the multiple cloning site of pGP704, creating plasmid pGP704_ ΔMCS, and then introduced the transposition functions of plasmid pML001 that we had used previously

(Himar1 transposase under control of P*tac-lac* and *lacI*^{q}) [17]. Into the resulting plasmid – pAH182_orig – we inserted a first experimental CRAfT sensor transposon that we had designed *in silico* and obtained from DNA synthesis by GeneART (Invitrogen; creating pAH182_CRAfT_v1). However, this transposon did not perform favorably in CRAfT experiments, so that we designed various other CRAfT sensors in order to generate a versatile CRAfT sensor. Finally, we created a CRAfT sensor module based on the kanamycin resistance cassette of well-known plasmid pKD13 (the lambda red recombineering template plasmid used in the KEIO collection [5, 24]) which was disrupted by a floxed spectinomycin resistance cassette of plasmid pGB2 [25]. For this purpose, we cloned the spectinomycin resistance cassette of pGB2 with flanking *loxP* sites into the Tn5 aminoglycoside phosphotransferase gene of pKD13 in a way that 1) the start codon of the latter gene was separated from the rest of the open reading frame (ORF) and 2) Cre / *loxP* recombination would restore kanamycin resistance by reconstituting the aminoglycoside phosphotransferase ORF with a short peptide of the *loxP* scar in frame (plasmid pAH195_v1). Such a setup of disruption and restoration of resistance cassettes has been repeatedly used for CRAfT [20, 26]. The resulting CRAfT sensor module was cloned into pAH182_CRAfT_v1 – creating plasmid pAH182_CRAfT_v8.1 or simply pAH182_CRAfT – and performed very well in CRAfT assays with high sensitivity and very low background (see, e.g., Fig 5B). Similar to the setup of pML001, plasmid pAH182_CRAfT has been designed in a way that the transposon is flanked by XbaI restriction sites on the proximal sides of the Himar1 inverted repeats, enabling easy replacement of the transposon cargo with any other DNA sequence. The molecular biology details of the construction of pAH182_CRAfT are summarized in Table A below.

Construction of the pAH183 series of plasmids

For the expression of Cre-fusions of candidate proteins in the donors of CRAfT assays we decided to use a non-mobilizable variant of broad-host range plasmid pBZ485, pBZ485_Δdtr. We first cloned the *cre* recombinase gene of bacteriophage P1 into this backbone, followed by DNA sequence encoding a

flexible G4S (4x glycine, 1x serine) linker [27] (creating plasmid pAH183_cre). This backbone was then modified by inserting candidate protein fusions to the C-terminal end of Cre-G4S or, in order to generate an empty vector for control experiments, by removing the *ccdB* cassette and adding a stop codon to Cre-G4S (creating pAH183_cre(only)). The different plasmids of the pAH183 series and the details of their construction are summarized in Table A below.

Table A. List and construction of all plasmids used in this study

Plasmid	Description	internal designation	Primers used for insert PCR		Template for PCR	Details on molecular cloning			Source / Reference
			fwd	rev		restriction enzymes (insert)	vector backbone	restriction enzymes (vector)	
**pME6032**	*E. coli* / *Pseudomonas* shuttle vector								our laboratory collection [28]
**pNDM220**	P*lac* plasmid with mini-R1 origin of replication (*ori*)								our laboratory collection [29]
…_btrficT	P*lac::btrficT* in pNDM220	pAH154_btrficT	prAH523	prAH524	*B. tribocorum* CIP105476	KpnI / XhoI	pNDM220	KpnI / XhoI	this study
…_btrficT(H136A)	P*lac::btrficT* in pNDM220 with H136A mutation in FIC domain active site	pAH154_btrficT_H136A	prAH443	prAH444	pAH154_btrficTt	n.a. (template plasmid modified by method of Liu and Naismith [30])			this study
**pBAD33**	P*ara* expression plasmid with p15A *ori*								our laboratory collection [31]
…_btrficA	P*ara::btrficA* in pBAD33	pAH153_btrficA	prAH498	prAH499	*B. tribocorum* CIP105476	KpnI / HindIII	pBAD33	KpnI / HindIII	this study
**plasmid F**									strain W1872 [32]
pAH204	F *tn1000::(camR)*; *camR* amplified from pMMB206 with suitable homologies and recombineered onto plasmid F		prAH1441	prAH1442	pMMB206	n.a. (template plasmid modified by method of Liu and Naismith [30])			this study
**pRSFDuet-1**	P*T7* plasmid for protein overexpression								Novagen
pPE0017	P*T7:: His6-vbhT* in MCS1 of pRSFDuet-1								our laboratory collection [33]
pPE0034	P*T7:: His6-vbhT_H136A* in MCS1 of pRSFDuet-1								our laboratory collection [33]
pPE0021	P*T7:: His6-vbhT* in MCS1 and P*T7::HA-vbhA* in MCS2 of pRSFDuet-1								our laboratory collection [33]
paAH134a	P*T7::His6-btrficT* in MCS1 of pRSFDuet-1		prAH404	prAH405	*B. tribocorum* CIP105476	PstI / NotI	pRSFDuet-1	PstI / NotI	this study
pAH134b	P*T7::His6-btrficT* in MCS1 and (marginally expressed) P*T7::HA-btrficA* in MCS2 of pRSFDuet-1		prAH402	prAH403	*B. tribocorum* CIP105476	BglII / XhoI	pAH134a	BglII / XhoI	this study
pAH134b_H136A	P*T7::His6-btrficT_H136A* in MCS1 and (marginally expressed) P*T7::HA-btrficA* in MCS2 of pRSFDuet-1		prAH443	prAH444	pAH134b	n.a. (template plasmid modified by method of Liu and Naismith [30])			this study
pAH134b_cor2	P*T7::His6-btrficT* in MCS1 and P*T7::HA-btrficA* in MCS2 of pRSFDuet-1		prAH547	prAH548	pAH134b	n.a. (template plasmid modified by method of Liu and Naismith [30])			this study
**pGex6p1**	P*lac* plasmid for protein overexpression of proteins tagged with glutathione S-transferase (GST)								GE Healthcare
pAH111	P*lac::GST-gyrB* (*E. coli*) in pGex6p1								our laboratory collection [1]
pAH119	P*lac::GST-parE* (*E. coli*) in pGex6p1								our laboratory collection [1]
**pCD341**	P*tac-lac* shuttle vector (*E. coli* / *Bartonella*) with RSF1010 backbone								our laboratory collection [7]
pMMB206	ancestor of pCD341 harboring a chloramphenicol resistance cassette (instead of kanamycin resistance)								our laboratory collection [34]
pCD353	pCD341 with P*tac-lac::gfpmut2*								our laboratory collection [7]
pCD353_v5	pCD353 with P*lac*(MQ5) instead of P*tac-lac*		prMQ1681	prMQ1682	pCD353	n.a. (template plasmid modified by method of Liu and Naismith [30])			this study
pBZ485a	pCD353_v5 in which *gfpmut2* was replaced with a *ccdB* cassette including a second MCS		prAH939	prAH940	*E. coli* W1872	XbaI / HindIII	pCD353_v5	XbaI / HindIII	this study

pBZ485a_Δdtr	pBZ485a in which the DNA processing and transfer (Dtr) region of RSF1010 was deleted		prAH1123	prAH1124	pBZ485a	n.a. (template plasmid modified by method of Liu and Naismith [30])			this study
pBZ485a_Δdtr_camR	variant of pBZ485a_Δdtr conferring chloramphenicol resistance instead of kanamycin resistance		prAH1238	prAH1239	pMMB206	NsiI / PstI	backbone of pBZ485a_Δdtr (amplified with prAH1240 / prAH1241)	NsiI / PstI	
**pBZ485**	new *E. coli* / *Bartonella* shuttle vector based on pCD341 with P*lac*(MQ5); RP4 *oriT*	pBZ485a_v2	prAH1267	prAH1268	pBZ485a_Δdtr	n.a. (template plasmid modified by method of Liu and Naismith [30])			this study
pAH183_cre	*cre* with a C-terminal G4S (4x glycine, 1x serine) linker cloned into pBZ485a_Δdtr	pAH183_empty	prAH1117	prAH1118	P1*vir* lysate	EcoRI	pBZ485a_Δdtr	MfeI / EcoRI	this study
pAH183_cre(only)	deletion of *ccdB* cassette from pAH183_cre and introduction of a stop codon at the end of Cre-G4S		prAH1245	prAH1246	pAH183_cre	n.a. (template plasmid modified by method of Liu and Naismith [30])			this study
pAH183_traI(RP4)	Cre-TraI(RP4) relaxase fusion in pBZ485a_Δdtr		prAH1181	prAH1182	JKE201	EcoRI / XhoI	pAH183_cre	EcoRI / XhoI	this study
pAH183_traI(F)	Cre-TraI(F) relaxase fusion in pBZ485a_Δdtr		prAH1435	prAH1436	*E. coli* W1872	EcoRI / XhoI	pAH183_cre	EcoRI / XhoI	this study
pAH183_traA	Cre-TraA(pVbh_BscR1) relaxase fusion in pBZ485a_Δdtr		prAH1247	prAH1248	*B. schoenbuchensis* R1	EcoRI / XhoI	pAH183_cre	EcoRI / XhoI	this study
pAH183_vbhT	Cre-VbhT(H136A)(pVbh_BscR1) toxin fusion in pBZ485a_Δdtr		prAH1249	prAH1248	pPE0034	EcoRI / XhoI	pAH183_cre	EcoRI / XhoI	this study
**pTR1000**	*Bartonella* suicide plasmid with *kanR* / *rpsL* double-selectable cassette for scarless deletions								our laboratory collection [11]
pPE3001	pTR1000 with homology sites to delete *vbhB4* of pVbh_BscR1 (homology regions amplified separately and then fused by SOEing PCR)		prPE506 / prPE507 for homology 1, prPE508 / prPE509 for homology 2; SOE PCR with prPE506 / prPE509		*B. schoenbuchensis* R1	BamHI	pTR1000	BamHI	this study
pPE3007	pTR1000 with homology sites to delete an N-terminal region of pVbh_BscR1 *traA* (homology regions amplified separately and then fused by SOEing PCR)		prPE609 / prPE511 for homology 1, prPE610 / prPE611 for homology 2; SOE PCR with prPE609 / prPE611		*B. schoenbuchensis* R1	BamHI	pTR1000	BamHI	this study
pML001	*Bartonella* suicide plasmid to deliver a Himar1 transposon carrying R6K *ori*, RP4 *oriT*, and gentamicin resistance								our laboratory collection [17]
pKD13	template plasmid encoding a kanamycin resistance cassette (designed for lambda red recombineering)								our laboratory collection [5]
pJM05	*Bartonella* suicide plasmid with *kanR* / *sacB* double-selectable cassette for scarless deletions								our laboratory collection [35]
**pGP704**	suicide vector with R6K *ori*, RP4 *oriT*, and *ampR*								our laboratory collection [22]
pGP704_ΔMCS	derivative of pGP704 in which its MCS has been deleted		prAH1113	prAH1114	pGP704	n.a. (template plasmid modified by method of Liu and Naismith [30])			this study
pAH182_orig	pGP704 backbone carrying transposition functions of pML001 (cut out as a fragment using PstI / KpnI)	pAH182	prAH1115	prAH1116	pGP704_Δ	SbfI / KpnI	pML001	PstI / KpnI	this study
pAH182_CRAfT_v1	pAH182_orig carrying a first transposon with an experimental CRAfT sensor module		transposon with an initial, experimental CRAfT sensor module (synthesized by GeneART, Invitrogen)			KpnI	pAH182_orig	KpnI	this study
pAH195_v1	spectinomycin resistance cassette with flanking *loxP* sites placed within the kanamycin resistance cassette of pKD13 (see dedicated section of S1 Text)		prAH1302	prAH1303	pAH182_CRAfT_v1 (floxed *spectR*)	EcoRI	pKD13 backbone amplified with prAH1300 / prAH1301	EcoRI / XhoI	this study
pAH182_CRAfT	pAH182 in which the actual Himar1 transposon in pAH182_CRAfT_v1 has been replaced with an optimized CRAfT sensor module	pAH182_CRAfT_v8.1	prAH1321	prAH1322	stacked *kanR* / *spectR* of pAH195_v1	SbfI / NotI	pAH182_CRAfT_v1	SbfI / NotI	this study
pAH188_orig	derivative pME6032 with MCS / expression functions and *camR* cassette of pBZ485a_Δdtr_camR		prAH1239	prAH1242	pBZ485a_Δdtr_camR	SalI / PstI	backbone of pME6032	PstI / XhoI	this study

							amplified with prAH1243 / prAH1244		
pAH188_oriT	pAH188_orig with RP4 *oriT* (analogous to step from pBZ485a_Δdtr_camR to pBZ485)		prAH1267	prAH1268	pAH188_orig	n.a. (template plasmid modified by method of Liu and Naismith [30])			this study
**pAH188**	pAH188_oriT without *ccdB* cassette	pAH188_oriT_plain	prAH1393	prAH1394	pAH188_oriT	n.a. (template plasmid modified by method of Liu and Naismith [30])			this study

References

1. Harms A, Stanger FV, Scheu PD, de Jong IG, Goepfert A, Glatter T, et al. Adenylylation of Gyrase and Topo IV by FicT Toxins Disrupts Bacterial DNA Topology. Cell Rep. 2015;12(9):1497-507. doi: 10.1016/j.celrep.2015.07.056. PubMed PMID: 26299961.

2. Ferrieres L, Hemery G, Nham T, Guerout AM, Mazel D, Beloin C, et al. Silent mischief: bacteriophage Mu insertions contaminate products of *Escherichia coli* random mutagenesis performed using suicidal transposon delivery plasmids mobilized by broad-host-range RP4 conjugative machinery. J Bacteriol. 2010;192(24):6418-27. Epub 2010/10/12. doi: 10.1128/jb.00621-10. PubMed PMID: 20935093; PubMed Central PMCID: PMCPmc3008518.

3. Monk IR, Tree JJ, Howden BP, Stinear TP, Foster TJ. Complete Bypass of Restriction Systems for Major *Staphylococcus aureus* Lineages. MBio. 2015;6(3):e00308-15. doi: 10.1128/mBio.00308-15. PubMed PMID: 26015493; PubMed Central PMCID: PMCPMC4447248.

4. Zhang G, Wang W, Deng A, Sun Z, Zhang Y, Liang Y, et al. A mimicking-of-DNA-methylation-patterns pipeline for overcoming the restriction barrier of bacteria. PLoS Genet. 2012;8(9):e1002987. doi: 10.1371/journal.pgen.1002987. PubMed PMID: 23028379; PubMed Central PMCID: PMCPMC3459991.

5. Datsenko KA, Wanner BL. One-step inactivation of chromosomal genes in *Escherichia coli* K-12 using PCR products. Proc Natl Acad Sci USA. 2000;97(12):6640-5. doi: 10.1073/pnas.120163297. PubMed PMID: 10829079; PubMed Central PMCID: PMC18686.

6. Shaw KJ, Rather PN, Hare RS, Miller GH. Molecular genetics of aminoglycoside resistance genes and familial relationships of the aminoglycoside-modifying enzymes. Microbiol Rev. 1993;57(1):138-63. PubMed PMID: 8385262; PubMed Central PMCID: PMCPMC372903.

7. Dehio M, Knorre A, Lanz C, Dehio C. Construction of versatile high-level expression vectors for *Bartonella henselae* and the use of green fluorescent protein as a new expression marker. Gene. 1998;215(2):223-9. Epub 1998/08/26. PubMed PMID: 9714815.

8. Cascales E, Atmakuri K, Liu Z, Binns AN, Christie PJ. *Agrobacterium tumefaciens* oncogenic suppressors inhibit T-DNA and VirE2 protein substrate binding to the VirD4 coupling protein. Mol Microbiol. 2005;58(2):565-79. Epub 2005/10/01. doi: 10.1111/j.1365-2958.2005.04852.x. PubMed PMID: 16194240; PubMed Central PMCID: PMCPmc2749481.

9. Segal G, Shuman HA. Intracellular multiplication and human macrophage killing by *Legionella pneumophila* are inhibited by conjugal components of IncQ plasmid RSF1010. Mol Microbiol. 1998;30(1):197-208. Epub 1998/10/24. PubMed PMID: 9786196.

10. Chen L, Chen Y, Wood DW, Nester EW. A new type IV secretion system promotes conjugal transfer in *Agrobacterium tumefaciens*. J Bacteriol. 2002;184(17):4838-45. Epub 2002/08/10. PubMed PMID: 12169609; PubMed Central PMCID: PMCPmc135267.

11. Schulein R, Guye P, Rhomberg TA, Schmid MC, Schroder G, Vergunst AC, et al. A bipartite signal mediates the transfer of type IV secretion substrates of *Bartonella henselae* into human cells. Proc Natl Acad Sci USA. 2005;102(3):856-61. doi: 10.1073/pnas.0406796102. PubMed PMID: 15642951; PubMed Central PMCID: PMC545523.

12. Harms A, Segers FH, Quebatte M, Mistl C, Manfredi P, Korner J, et al. Evolutionary dynamics of pathoadaptation revealed by three independent acquisitions of the VirB/D4 type IV secretion system in *Bartonella*. Genome Biol Evol. 2017. doi: 10.1093/gbe/evx042. PubMed PMID: 28338931.

13. Quebatte M, Dehio M, Tropel D, Basler A, Toller I, Raddatz G, et al. The BatR/BatS two-component regulatory system controls the adaptive response of *Bartonella henselae* during human endothelial cell infection. J Bacteriol. 2010;192(13):3352-67. doi: 10.1128/JB.01676-09. PubMed PMID: 20418395; PubMed Central PMCID: PMCPMC2897681.

14. Bernard P, Gabant P, Bahassi EM, Couturier M. Positive-selection vectors using the F plasmid *ccdB* killer gene. Gene. 1994;148(1):71-4. Epub 1994/10/11. PubMed PMID: 7926841.

15. Meyer R. Replication and conjugative mobilization of broad host-range IncQ plasmids. Plasmid. 2009;62(2):57-70. doi: 10.1016/j.plasmid.2009.05.001. PubMed PMID: 19465049; PubMed Central PMCID: PMCPMC2752045.

16. Henderson D, Meyer RJ. The primase of broad-host-range plasmid R1162 is active in conjugal transfer. J Bacteriol. 1996;178(23):6888-94. Epub 1996/12/01. PubMed PMID: 8955311; PubMed Central PMCID: PMCPmc178590.

17. Quebatte M, Dick MS, Kaever V, Schmidt A, Dehio C. Dual input control: activation of the *Bartonella henselae* VirB/D4 type IV secretion system by the stringent sigma factor RpoH1 and the BatR/BatS two-component system. Mol Microbiol. 2013;90(4):756-75. doi: 10.1111/mmi.12396. PubMed PMID: 24033511.

18. den Dulk-Ras A, Vergunst AC, Hooykaas PJ. Cre Reporter Assay for Translocation (CRAfT): a tool for the study of protein translocation into host cells. Methods Mol Biol. 2014;1197:103-21. Epub 08/31. doi: 10.1007/978-1-4939-1261-2_6. PubMed PMID: 25172277.

19. Vergunst AC, Schrammeijer B, den Dulk-Ras A, de Vlaam CM, Regensburg-Tuink TJ, Hooykaas PJ. VirB/D4-dependent protein translocation from *Agrobacterium* into plant cells. Science. 2000;290(5493):979-82. PubMed PMID: 11062129.

20. Lang S, Gruber K, Mihajlovic S, Arnold R, Gruber CJ, Steinlechner S, et al. Molecular recognition determinants for type IV secretion of diverse families of conjugative relaxases. Mol Microbiol. 2010;78(6):1539-55. doi: 10.1111/j.1365-2958.2010.07423.x. PubMed PMID: 21143323.

21. Kienesberger S, Schober Trummler C, Fauster A, Lang S, Sprenger H, Gorkiewicz G, et al. Interbacterial macromolecular transfer by the *Campylobacter fetus* subsp. *venerealis* type IV secretion system. J Bacteriol. 2011;193(3):744-58. doi: 10.1128/JB.00798-10. PubMed PMID: 21115658; PubMed Central PMCID: PMCPMC3021226.

22. Miller VL, Mekalanos JJ. A novel suicide vector and its use in construction of insertion mutations: osmoregulation of outer membrane proteins and virulence determinants in *Vibrio cholerae* requires *toxR*. J Bacteriol. 1988;170(6):2575-83. PubMed PMID: 2836362; PubMed Central PMCID: PMCPMC211174.

23. Kolter R, Inuzuka M, Helinski DR. Trans-complementation-dependent replication of a low molecular weight origin fragment from plasmid R6K. Cell. 1978;15(4):1199-208. PubMed PMID: 728998.

24. Baba T, Ara T, Hasegawa M, Takai Y, Okumura Y, Baba M, et al. Construction of *Escherichia coli* K-12 in-frame, single-gene knockout mutants: the Keio collection. Mol Syst Biol. 2006;2:2006 0008. doi: 10.1038/msb4100050. PubMed PMID: 16738554; PubMed Central PMCID: PMC1681482.

25. Churchward G, Belin D, Nagamine Y. A pSC101-derived plasmid which shows no sequence homology to other commonly used cloning vectors. Gene. 1984;31(1-3):165-71. PubMed PMID: 6098521.

26. Luo ZQ, Isberg RR. Multiple substrates of the *Legionella pneumophila* Dot/Icm system identified by interbacterial protein transfer. Proc Natl Acad Sci U S A. 2004;101(3):841-6. doi: 10.1073/pnas.0304916101. PubMed PMID: 14715899; PubMed Central PMCID: PMCPMC321768.

27. Chen X, Zaro JL, Shen WC. Fusion protein linkers: property, design and functionality. Adv Drug Deliv Rev. 2013;65(10):1357-69. doi: 10.1016/j.addr.2012.09.039. PubMed PMID: 23026637; PubMed Central PMCID: PMCPMC3726540.

28. Heeb S, Blumer C, Haas D. Regulatory RNA as Mediator in GacA/RsmA-Dependent Global Control of Exoproduct Formation in *Pseudomonas fluorescens* CHA0. J Bacteriol. 2002;184(4):1046-56. doi: 10.1128/jb.184.4.1046-1056.2002.

29. Gotfredsen M, Gerdes K. The *Escherichia coli relBE* genes belong to a new toxin-antitoxin gene family. Mol Microbiol. 1998;29(4):1065-76. Epub 1998/10/10. PubMed PMID: 9767574.

30. Liu H, Naismith JH. An efficient one-step site-directed deletion, insertion, single and multiple-site plasmid mutagenesis protocol. BMC Biotechnol. 2008;8:91. doi: 10.1186/1472-6750-8-91. PubMed PMID: 19055817; PubMed Central PMCID: PMC2629768.

31. Guzman LM, Belin D, Carson MJ, Beckwith J. Tight regulation, modulation, and high-level expression by vectors containing the arabinose PBAD promoter. J Bacteriol. 1995;177(14):4121-30. Epub 1995/07/01. PubMed PMID: 7608087; PubMed Central PMCID: PMC177145.

32. Lederberg EM, Lederberg J. Genetic Studies of Lysogenicity in *Escherichia Coli*. Genetics. 1953;38(1):51-64. PubMed PMID: 17247421; PubMed Central PMCID: PMCPMC1209586.

33. Engel P, Goepfert A, Stanger FV, Harms A, Schmidt A, Schirmer T, et al. Adenylylation control by intra- or intermolecular active-site obstruction in Fic proteins. Nature. 2012;482(7383):107-10. Epub 01/24. doi: 10.1038/nature10729. PubMed PMID: 22266942.

34. Morales VM, Backman A, Bagdasarian M. A series of wide-host-range low-copy-number vectors that allow direct screening for recombinants. Gene. 1991;97(1):39-47. PubMed PMID: 1847347.

35. MacKichan JK, Gerns HL, Chen YT, Zhang P, Koehler JE. A SacB mutagenesis strategy reveals that the *Bartonella quintana* variably expressed outer membrane proteins are required for bloodstream infection of the host. Infect Immun. 2008;76(2):788-95. doi: 10.1128/IAI.01174-07. PubMed PMID: 18070893; PubMed Central PMCID: PMC2223462.
